# Raman Spectroscopy Can Identify Acute and Persistent Biochemical Changes in Leukocytes From Patients With COVID‐19 and Non‐COVID‐19‐Associated Sepsis

**DOI:** 10.1002/biot.70105

**Published:** 2025-09-01

**Authors:** Anuradha Ramoji, Philipp Baumbach, Oleg Ryabchykov, Aikaterini Pistiki, Jan Rueger, David Vasquez Pinzon, Anja Silge, Stefanie Deinhardt‐Emmer, Iwan W. Schie, Karina Weber, Charles Neu, Ute Neugebauer, Michael Kiehntopf, Thomas Bocklitz, Juergen Popp, Sina M. Coldewey

**Affiliations:** ^1^ Institute of Physical Chemistry and Abbe Center of Photonics, Friedrich Schiller University Jena Member of the Leibniz Centre for Photonics in Infection Research (LPI) Jena Germany; ^2^ Leibniz Institute of Photonic Technology – Member of the Research Alliance “Leibniz Health Technologies” Member of the Leibniz Centre For Photonics in Infection Research (LPI) Jena Germany; ^3^ Department of Anesthesiology and Intensive Care, Jena University Hospital Friedrich‐Schiller‐University Jena Germany; ^4^ Septomics Research Centre, Jena University Hospital Friedrich‐Schiller‐University Jena Jena Germany; ^5^ Institute of Medical Microbiology, Jena University Hospital Friedrich‐Schiller‐University Jena Jena Germany; ^6^ Department of Medical Engineering and Biotechnology University of Applied Sciences Jena Jena Germany; ^7^ Center for Sepsis Control and Care Jena University Hospital Friedrich‐Schiller‐University Jena Jena Germany; ^8^ Institute for Clinical Chemistry and Laboratory Diagnostics, Jena University Hospital Jena Germany; ^9^ Institute of Anesthesiology and Perioperative Medicine, University Hospital Zurich, University of Zurich Zurich Switzerland

**Keywords:** COVID‐19, critical care, critical illness, intensive care unit, leukocytes, Raman spectroscopy, sepsis

## Abstract

Sepsis remains a major clinical challenge, often resulting in long‐term physiological and immunological disturbances. This study employed high‐throughput single‐cell Raman spectroscopy to analyze the biochemical profiles of peripheral blood leukocytes from patients with non‐COVID‐19 and COVID‐19‐associated sepsis. Leukocytes were assessed at multiple timepoints, including the acute phase (Days 3 and 7 after sepsis onset) and late recovery phase (6 and 12 months after sepsis onset). Raman spectroscopic profiles of leukocytes showed clear separation between healthy controls and sepsis patients during the acute phase with high balanced accuracy (BAcc: 95%–98%). Spectral differences between acute and recovery phases (BAcc: 84%–97%) and between recovery‐phase leukocytes and those from healthy controls (BAcc: 81%–90%) were also observed, indicating long‐lasting molecular alterations. Furthermore, distinct profiles were identified between non‐COVID‐19 and COVID‐19‐associated sepsis during the acute phase (BAcc: 65%–71%) and in the late‐recovery phase (BAcc: 71%–83%). These findings demonstrate that Raman spectroscopy enables label‐free, high‐throughput profiling of leukocyte biochemistry across the sepsis trajectory. This suggests that Raman spectroscopy is a promising tool for high‐throughput screening, offering insights into the biomolecular changes in sepsis and providing a diagnostic platform to differentiate between sepsis etiologies, a significant advancement in the field of sepsis diagnostics.

## Graphical Abstract and Lay Summary

High‐throughput single‐cell Raman spectroscopy revealed distinct biochemical profiles in leukocytes from patients with sepsis, distinguishing them from healthy controls with high accuracy. Persistent biomolecular alterations were observed up to 12 months after sepsis onset, indicating long‐term immune dysregulation. Differences in leukocyte profiles between patients with COVID‐19 and non‐COVID‐19‐associated sepsis suggest potential for etiology‐specific diagnostics.

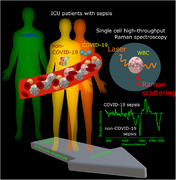

## 1 Introduction

Sepsis is defined as a life‐threatening organ dysfunction caused by a dysregulated host response to infection [1]. It is a significant medical concern, particularly among critically ill patients in intensive care units (ICUs). Although the supportive treatment of patients with sepsis has improved, the mortality rate remains high [1, 2, 3, 4]. The main reason is the lack of early, sensitive, and specific diagnostic methods and appropriate therapeutic measures [5, 6, 7, 8]. This implies novel methods for early recognition of sepsis in patients, and the right therapeutic approaches are needed.

To address this problem, several markers have been developed, including those based on clinical signs and circulating small peptides‐based biomarkers that are over‐produced by the leukocytes during their interaction with the invading infectious agent. Some patients exhibit over‐reactive circulating leukocytes that remain persistent compared to others, and in many cases, clinical signs are not fulminant, leading to difficulties in diagnosis. After a sepsis episode, mortality and morbidity can increase for years, and only a few patients return to normality [9, 10, 11, 12, 13]. These health restrictions are summarized as post‐intensive care syndrome and are associated with a reduction in health‐related quality of life [14, 15].

The 2019 coronavirus disease pandemic (COVID‐19), which has resulted in a high incidence of critically ill intensive care patients with SARS‐CoV‐2‐related sepsis worldwide [16], has demonstrated the necessity for differentiated diagnosis and treatment of the various phenotypes of sepsis [17]. The diverse range of symptoms exhibited by patients with sepsis presents a significant challenge to the implementation of novel theragnostic approaches [13]. However, early diagnosis and precise immunological phenotyping are essential for selecting and implementing effective personalized treatment strategies in this highly diverse group of patients with sepsis [18]. Early assessment of patient response not only helps in adjusting treatments for better long‐term sepsis outcomes but also aids in better characterizing the patients and their unique immune response. [5, 6, 16, 19, 20, 21]

In response to the COVID‐19 outbreak, new optical‐based methods have emerged that use the biochemical signature of the samples to identify the cause of infection or inflammation and assess the patient's immune status [22, 23, 24, 25, 26]. Raman spectroscopy, an analytical technique that provides information on the molecular composition of a sample, has gained prominence as a diagnostic tool [27, 28, 29, 30]. Several studies have been published on the use of Raman spectroscopy to detect infections and their various causes, including COVID‐19 detection from various body fluids, immune cells, and tissues [29, 31, 32]. However, the application of Raman spectroscopy for cell diagnostics has often relied on commercial Raman microscopes, where acquisition speed is primarily limited by system throughput and hardware design. These limitations make conventional systems time‐consuming and less suited for screening large patient cohorts. Schie et al. implemented high‐throughput Raman spectroscopy, enabling the screening of approximately 2000 cells per hour, which has streamlined the use of Raman spectroscopy in label‐free cell diagnostics for clinical investigations [33]. This study aims to translate this technology into clinical workflows, potentially impacting patient stratification through the generation of robust and statistically meaningful spectroscopic datasets.

Previously, for the first time, we showed Raman spectroscopy analysis of leukocytes can be performed from as little as 500 µL of blood for sepsis diagnosis in critically ill patients [34]. The main objectives of the current study are to utilize high‐throughput screening Raman spectroscopy in conjunction with machine learning algorithm: (1) to longitudinally monitor changes in the biochemical profile of leukocytes from critically ill patients with sepsis from the acute to the late recovery phase of disease, (2) differentiate between patients with non‐COVID‐19 and COVID‐19‐associated sepsis, and (3) investigate the effect of sex and age as potential confounders of Raman spectroscopy.

## 2 Materials and Methods

### 2.1 Study Design and Data Collection

Data were collected within the prospective monocentric cohort study “Identification of cardiovascular and molecular prognostic factors for the medium‐term and long‐term outcomes of sepsis” (ICROS) at the Jena University Hospital (JUH). The details of the study are outlined in the published study protocol [35]. In brief, patients with sepsis were enrolled in the ICUs of the JUH if they met the Sepsis‐3 criteria [11]. The main exclusion criteria for patients with sepsis and healthy controls comprised significant heart condition or end‐stage kidney or liver disease, pregnancy or breastfeeding, or therapy limitation or do‐not‐resuscitate order. In addition, healthy controls should not have recently suffered from sepsis (≤ 8 months) or critical illness (≤ 6 months).

**FIGURE 1:**
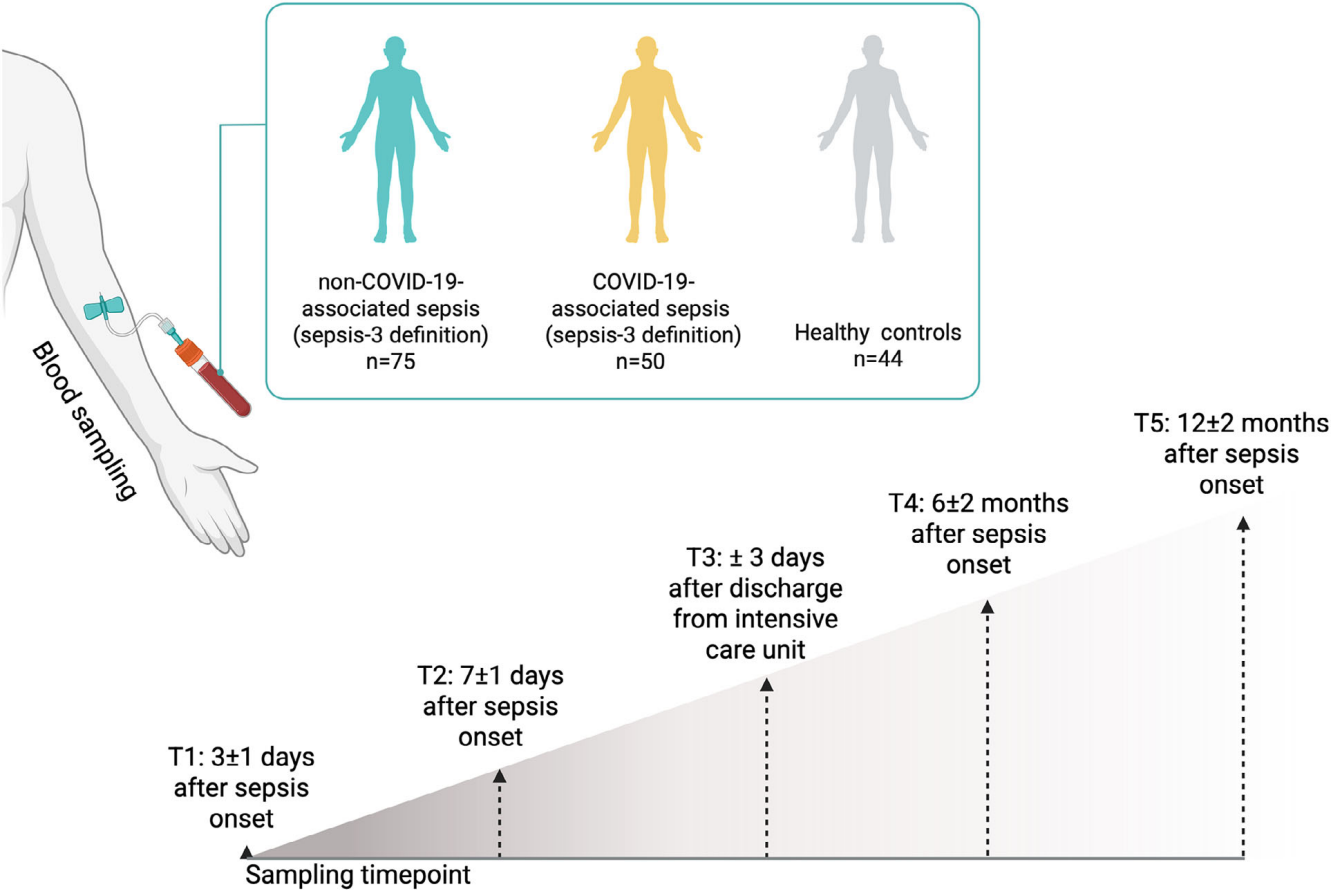
Schematic diagram of the clinical study design. Created in BioRender. Silge, A. (2025) https://BioRender.com/bvnjjgg.

In the acute and subacute phases of sepsis, data were collected 3 ± 1 days (*T*_1_) and 7 ± 1 days (*T*_2_) after sepsis onset. Additionally, data were collected in the early (*T*_3_: ICU discharge ± 3 days) and the late recovery phase (*T*_4_: 6 ± 2 months and *T*_5_: 12 ± 2 months after sepsis onset). For healthy controls, we collected data at one study visit in the outpatient facilities of the Department of Anesthesiology and Intensive Care Medicine of the JUH. A schematic diagram for sample collection is presented in Figure 1. In the analysis, we report results for the patients with sepsis (both non‐COVID‐19‐associated and COVID‐19‐associated sepsis) and healthy controls. Additionally, patients with sepsis were stratified by COVID‐19 status (non‐COVID‐19‐ vs. COVID‐19‐associated sepsis).

### 2.2 Ethics Approval

The study is in accordance with the Declaration of Helsinki and was approved by the Ethics Committee of the Friedrich Schiller University Jena (5276‐09/17, October 10 2017). It is registered at the German Clinical Trials Register (DRKS00013347) and Clinical Trials.gov (NCT03620409).

### 2.3 High‐Throughput Raman Spectroscopy

Leukocytes were isolated from 500 µL of full blood (EDTA) and investigated within 48 h after collection (see Supporting Information) using an in‐house developed high‐throughput screening (HTS) Raman device [33], allowing the highly efficient characterization of ∼1500 cells/patient within 1 h. Briefly, the HTS Raman system is an upright Raman microscope equipped with a 785 nm single‐mode excitation laser with a power of 100 mW at the plane of the objective. The Raman scattered light was collected with a laser exposure of 1 s from the cells settled on a calcium fluoride slide and immersed in distilled water via a 60× objective (NA 1.0, Nikon). The remaining blood was used for routine clinical analysis.

### 2.4 Statistical Analysis

#### 2.4.1 General Descriptive Analyses

We report median, first and third quartiles, or mean and standard deviation for continuous variables. In the case of categorical variables, we report absolute (*n*) and relative frequencies (%). Differences in continuous variables (age, body height, weight, and BMI) between healthy controls and patients with sepsis were tested with *t*‐tests. Sex differences were tested with a chi‐squared test.

#### 2.4.2 Raman Spectroscopy Marker

The Raman spectra of the leukocytes were preprocessed to make the Raman data collected from different cells and patients comparable [36, 37] (see Appendix SA). The preprocessed Raman spectra of the leukocytes were subjected to partial least‐squares discriminant analysis (PLS‐DA). Different binary classification models were built for each type of model response as follows:

1. Sex (male vs. female) and age (< 65 years vs. ≥ 65 years) were investigated to analyze the impact of demographic factors at *T*_1_.
2. Raman spectra of healthy controls versus patients with non‐COVID‐19‐associated sepsis, COVID‐19‐associated sepsis, and for both etiologies were analyzed to assess the sepsis‐induced leukocyte changes in the acute (*T*_1_), subacute (*T*_2_), and late recovery phases of sepsis (*T*_4_, *T*_5_).
3. Raman spectra of patients with non‐COVID‐19‐ versus COVID‐19‐associated sepsis were analyzed for potential COVID‐19 specific leukocyte changes in the acute (*T*_1_), subacute (*T*_2_), and late recovery phases of sepsis (*T*_4_, *T*_5_).
4. Raman spectra of the acute phase of sepsis versus later phases of sepsis (*T*_1_ vs. *T*_3_, *T*_1_ vs. *T*_4_, and *T*_1_ vs. *T*_5_) were analyzed to assess longitudinal changes during the recovery phase.

To balance the contribution from single patients onto the model and to improve the signal‐to‐noise ratio, the spectra collected from every single patient were divided into five chunks, and an average spectrum within each chunk was calculated, thus resulting in five aggregated spectra for each patient. To obtain a single prediction per patient, a major vote scheme was applied per patient to the predictions obtained for aggregated spectra. For balancing the different patient numbers per class, a canonical PLS algorithm [38, 39] that permits the weighting of the data was applied. The Raman model was evaluated using leave‐one‐patient‐out cross‐validation, which made it possible to generate independent predictions based on Raman spectra for each patient. For each Raman model, sensitivity, specificity, and balanced accuracy (BAcc) were calculated. The BAcc was calculated as an average of the sensitivity and specificity of the model. In addition, Cohen's kappa values were calculated to assess the reliability of the Raman models. The kappa values can be interpreted as follows: values ≤ 0 no agreement, 0.01–0.20 as none to slight, 0.21–0.40 as fair, 0.41–0.60 as moderate, 0.61–0.80 as substantial, and 0.81–1.00 as almost perfect agreement [40]. By analogy to Cohen's kappa interpretation, we interpret the balanced accuracy values for 2‐class models as follows: 0.6 and below as none to slight agreement, 0.61–0.70 as fair, 0.71–0.80 as moderate, 0.81–0.90 as substantial, and over 0.9 as almost perfect agreement.

## 3 Results

To investigate the ability of Raman spectroscopy‐based differentiation of patients with non‐COVID‐19‐associated sepsis and COVID‐19‐associated sepsis and healthy volunteers, peripheral blood leukocytes were analyzed (Figure 1). In total, 75 patients with non‐COVID‐19‐associated sepsis, 50 patients with COVID‐19‐associated sepsis, and 44 healthy controls were available for the analyses. Table S1 gives a detailed overview of the number of subjects for all time points.

### 3.1 Demographic and Clinical Characterization

Table 1 summarizes the demographic and clinical characteristics of the patient sample. In patients with non‐COVID‐19‐associated sepsis, pneumonia, and intra‐abdominal infections were the most common causes of sepsis. All patients with COVID‐19‐associated sepsis had SARS‐CoV‐2‐associated pneumonia or acute respiratory distress syndrome. Initial illness severity at sepsis onset tended to be higher in patients with non‐COVID‐19‐ versus COVID‐19‐associated sepsis. Patients with non‐COVID‐19‐associated sepsis tended to have a longer length of stay in the ICU and hospital. The majority of patients in both groups received mechanical ventilation during the ICU stay.

**TABLE 1:** Characterization of the patient sample.

		Total (*n* = 125)			Non‐COVID‐19‐associated sepsis (*n* = 75)			COVID‐19‐associated sepsis (*n* = 50)		
Variable	Category	*n*	(%)	*n*_valid_	*n*	(%)	*n*_valid_	*n*	(%)	*n*_valid_
Sex	Female	42	(33.6)	125	27	(36.0)	75	15	(30.0)	50
Septic shock at sepsis onset	Yes	39	(31.7)	123	36	(48.0)	75	3	(6.3)	48
COVID‐19	Yes	50	(40.0)	125	0	(0.0)	75	50	(100.0)	50
Diabetes mellitus	Yes	35	(28.7)	122	22	(30.6)	72	13	(26.0)	50
Sepsis focus	Pneumonia	82	(65.6)	125	32	(42.7)	75	50	(100.0)	50
	Intra‐abdominal	30	(24.0)	125	30	(40.0)	75	0	(0.0)	50
	Urologic	12	(9.6)	125	12	(16.0)	75	0	(0.0)	50
	Bones/soft tissue	7	(5.6)	125	7	(9.3)	75	0	(0.0)	50
	Primary bacteremia	6	(4.8)	125	5	(6.7)	75	1	(2.0)	50
	Thoracic	4	(3.2)	125	4	(5.3)	75	0	(0.0)	50
	Gastrointestinal	3	(2.4)	125	2	(2.7)	75	1	(2.0)	50
	Respiratory (others)	2	(1.6)	125	1	(1.3)	75	1	(2.0)	50
	Wound infection	2	(1.6)	125	2	(2.7)	75	0	(0.0)	50
	Intracerebral	2	(1.6)	125	2	(2.7)	75	0	(0.0)	50
	Others	2	(1.6)	124	2	(2.7)	74	0	(0.0)	50
	Multiple foci	25	(20.0)	125	22	(29.3)	75	3	(6.0)	50
Mechanical ventilation in the ICU	Yes	100	(80.6)	124^b^	62	(83.8)	74^b^	38	(76.0)	50
Renal replacement therapy in the ICU	Yes	34	(27.4)	124^b^	23	(31.1)	74^b^	11	(22.0)	50
**Variable**	**Unit**	**Mean**	**(SD)**	***n*_valid_**	**Mean**	**(SD)**	***n*_valid_**	**Mean**	**(SD)**	***n*_valid_**
Age	years	62.4	(14.9)	125	61.0	(15.8)	75	64.6	(13.3)	50
Body height	cm	171.7	(10.6)	125	170.9	(10.6)	75	173.0	(10.5)	50
Body weight	kg	88.4	(20.5)	125	84.7	(19.7)	75	94.0	(20.4)	50
Body mass index	kg/m^2^	30.0	(6.7)	125	29.1	(6.8)	75	31.4	(6.4)	50
Charlson Comorbidity Index	0–37	1.8	(1.9)	125	2.2	(2.1)	75	1.2	(1.5)	50
SOFA^a^ at sepsis onset	0–24	7.6	(3.6)	125	9.2	(3.2)	75	5.3	(2.8)	50
**Variable**	**Unit**	**Median**	**[Q_1_–Q_3_]**	***n*_valid_**	**Median**	**[Q_1_–Q_3_]**	***n*_valid_**	**Median**	**[Q_1_–Q_3_]**	***n*_valid_**
Mechanical ventilation in the ICU	days	7	[2–18]	124^b^	6	[2–19]	74^b^	8	[2–18]	50
Renal replacement therapy in the ICU	days	0	[0–2]	124^b^	0	[0–3]	74^b^	0	[0–0]	50
Length of stay: ICU	days	10	[6–23]	124^b^	10	[4–23]	74^b^	9	[6–24]	50
Length of stay: hospital	days	27	[15–42]	124^b^	33	[21–54]	74^b^	20	[13–29]	50

*Note*: In the case of categorical variables, the table presents the absolute (*n*) and relative frequencies (percentage, %). For continuous variables, the table presents the mean and standard deviation (SD) or median, first (Q_1_), and third quartile (Q_3_). In addition, we report the number of patients with valid/non‐missing data points (*n*_valid_).

^a^ Sepsis‐related organ failure assessment score.

^b^ One patient revoked consent for study participation after the first study visit (*T*_1_, 3 ± 1 days after sepsis onset).

The mean age did not differ significantly between healthy controls (mean ± SD: 62.1 ± 15.3 years) and patients with sepsis. Compared to the patient sample, the proportion of females was lower in healthy controls (15.1%, *n* = 7/44; *Χ^2^* (4.1), *df* = 1, *p* = 0.042). In addition, the body height (176.6 ± 11.1 cm) was significantly higher, and body weight (88.4 ± 20.5 kg) and BMI (25.1 ± 3.1 kg/m^2^) were significantly lower in healthy controls compared to the patient sample (all *p* values < 0.05).

### 3.2 Raman Spectroscopy Analysis of the Peripheral Blood Leukocytes

#### 3.2.1 Age and Sex Are Weakly Associated With Raman Spectral Data of Peripheral Leukocytes

**FIGURE 2:**
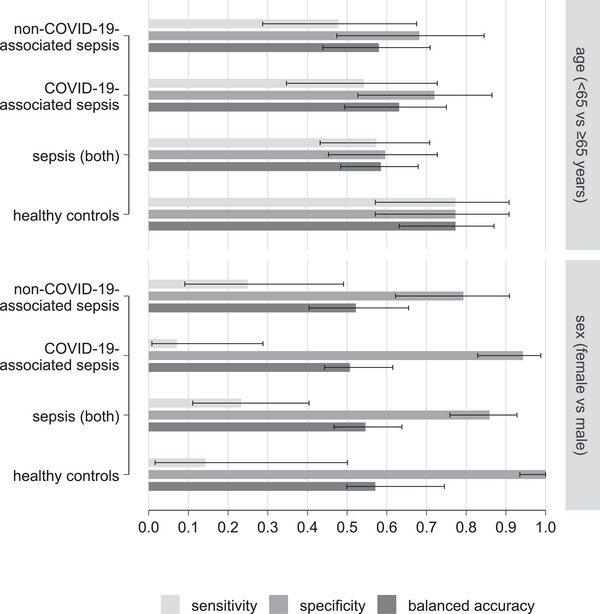
Association of age and sex with Raman spectral data of peripheral leukocytes from healthy controls and patients with sepsis (non‐COVID‐19‐associated sepsis, COVID‐19‐associated sepsis, and both). The bar graph shows sensitivity, specificity, and balanced accuracy (including 95% confidence intervals) obtained from Raman spectral‐based models using partial least‐squares discriminant analysis.

The results of the PLS‐DA models are summarized in Figure 2. Most of the models for age and sex demonstrated relatively poor performance with balanced accuracies (BAcc) below 60%. Cohen's kappa for the models ranged between 0.02 and 0.55 (slight to moderate agreement; see Table S2).

#### 3.2.2 Leukocyte Raman Spectral Data of Peripheral Leukocytes Effectively Differentiate Between Healthy Controls and Patients With Sepsis

**FIGURE 3:**
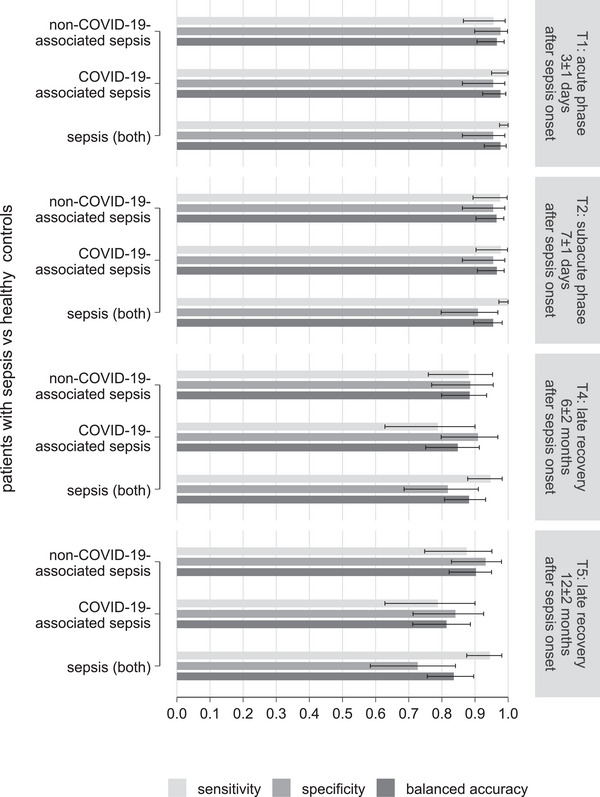
Raman spectroscopy differentiation between healthy controls and patients with sepsis based on the biochemical signature of peripheral leukocytes. The bar graph shows sensitivity, specificity, and balanced accuracy (including 95% confidence intervals) obtained from Raman spectral‐based models using partial least‐squares discriminant analysis for differentiation of healthy controls from patients with sepsis at different time points after the onset of sepsis. In addition, results for patients with non‐COVID‐19‐ and COVID‐19‐associated sepsis are presented. The results are shown for the acute phase and the recovery phase of sepsis. Loading coefficients of the models are shown in Figure S1.

Raman spectroscopy's diagnostic potential was evaluated by analyzing the leukocyte biochemical profile in the spectra of healthy volunteers and sepsis patients. The sensitivity, specificity, and BAcc for all PLS‐DA models to distinguish between patients with sepsis and healthy controls are summarized in Figure 3. The BAcc for the models differentiating between healthy controls and patients with sepsis (both non‐COVID‐19‐ and COVID‐19‐associated sepsis) showed a high performance in the acute (*T*_1_, 0.98) and subacute phase (*T*_2_, 0.95). The corresponding values of Cohen's *kappa* showed very good agreement between the actual and predicted group membership. The discriminatory power was in the same range when models were built separately for patients with non‐COVID‐19‐ or COVID‐19‐associated sepsis versus the healthy controls (Figure 3). The models for the late recovery phases (*T*_4_ and *T*_5_, Figure 3) were less accurate for all patients with sepsis (Cohen's kappa: 0.78 and 0.70, substantial agreement) as well as for patients with non‐COVID‐19‐associated sepsis (Cohen's kappa: 0.77 and 0.81) or COVID‐19‐associated sepsis (Cohen's kappa: 0.70 and 0.63). The values for sensitivity, specificity, BAcc, and Cohen's kappa for each model are listed in Table S3.

For applications in a clinical setting, estimating the sample size for training data is critically important. Unfortunately, in the case of multivariate data, this estimation is non‐trivial and highly dependent on model selection. One approach to define the required sample size is to determine the number of subjects needed to reach 95% of the Bayes error [41]. In the context of sepsis detection during the acute and subacute phases, the models achieved balanced accuracies of 95%, suggesting that the available sample size was sufficient for modeling with PLS‐DA.

#### 3.2.3 Raman Spectral Data of Peripheral Leukocytes Differentiate Between Patients With Non‐COVID‐19‐ Versus COVID‐19‐Associated Sepsis

**FIGURE 4:**
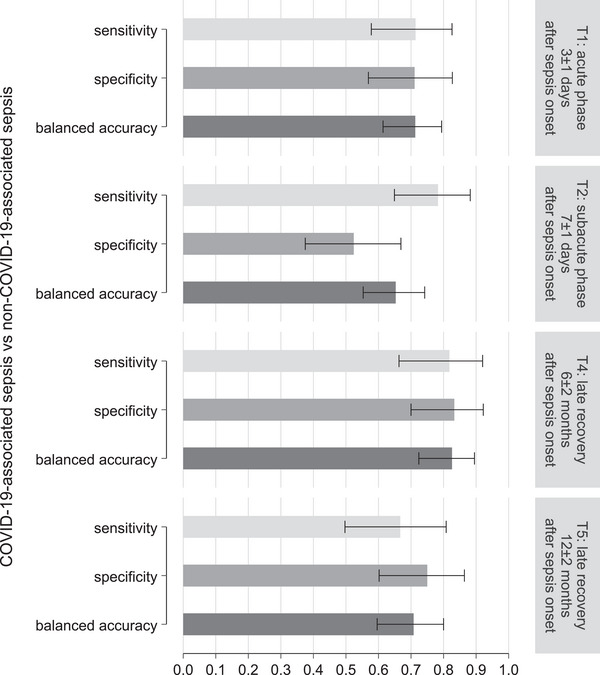
Raman spectroscopy differentiation between patients with non‐COVID‐19‐ versus COVID‐19‐associated sepsis based on the biochemical signature of peripheral leukocytes. The bar graph shows sensitivity, specificity, and balanced accuracy (including 95% confidence intervals) obtained from Raman spectral‐based models using partial least‐squares discriminant analysis. The results are presented for the acute phase and the recovery phase of sepsis. Loading coefficients of the models are shown in Figure S2.

Figure 4 summarizes the sensitivity, specificity, and BAcc obtained from different Raman models during the acute (*T*_1_), subacute (*T*_2_), and late recovery phases (*T*_4_ and *T*_5_). The values for BAcc ranged between 0.65 and 0.83, and the corresponding values of Cohen's kappa (range: 0.31–0.65) showed fair to substantial agreement between actual and predicted group membership. The discriminatory power was highest at the late recovery phase at 6 months (*T*_4_, BAcc: 0.83, Cohen's kappa: 0.65). The values for sensitivity, specificity, BAcc, and Cohen's kappa for each model are listed in Table S3.

#### 3.2.4 Raman Spectral Data of Peripheral Leukocytes Effectively Differentiate Between the Acute and Late Recovery Phase in Patients With Sepsis

**FIGURE 5:**
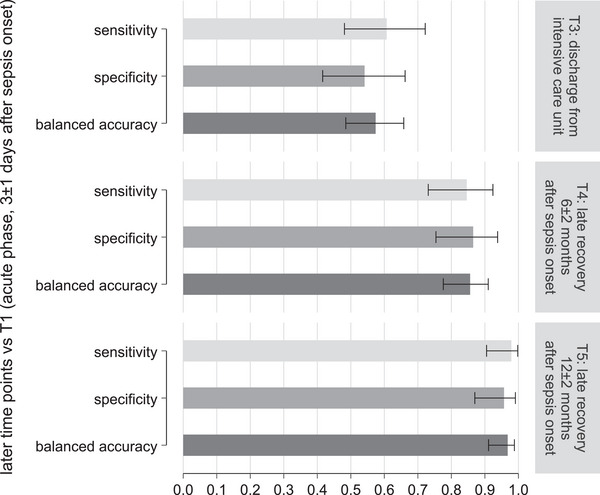
Raman spectroscopy investigations of longitudinal changes in peripheral leukocytes of patients with sepsis (patients with non‐COVID‐19‐ and COVID‐19‐associated sepsis). The bar graph shows sensitivity, specificity, and balanced accuracy (including 95% confidence intervals) obtained from Raman spectral‐based models using partial least‐squares discriminant analysis for detecting longitudinal changes in peripheral leukocytes. Results are presented for the early (discharge from the intensive care unit) and late recovery phase. Loading coefficients of the models are shown in Figure S3.

Figure 5 summarizes the sensitivity, specificity, and BAcc for the models in all patients with sepsis (non‐COVID‐19‐ and COVID‐19‐associated sepsis). The discriminatory power of the models increased from the early recovery phase (*T*_3_, BAcc: 0.57) to the late recovery phase at 6 months (*T*_4_, BAcc: 0.86) and 12 months (*T*_5_, BAcc: 0.97) after sepsis onset. In terms of Cohen's kappa, the agreement of actual and predicted time point increased from the early recovery phase (*T*_3_, 0.15) to late recovery phases at 6 and 12 months (*T*_4_, 0.71; *T*_5_, 0.94). The results were in a similar range for patients with non‐COVID‐19‐ or COVID‐19‐associated sepsis (see Table S3 and Figure S4).

## 4 Discussion

In the current study, we analyzed the chemical signature of peripheral blood leukocytes using high‐throughput Raman spectroscopy in the long‐term trajectory of critically ill patients with non‐COVID‐19‐ and COVID‐19‐associated sepsis. Raman spectral data were subjected to partial least‐squares discriminant analysis. In the first set of models, we analyzed the discriminative power of demographic factors (age and sex) on the Raman spectra data, which was relatively low. The second set of Raman models was built to discriminate between healthy volunteers and patients with non‐COVID‐19‐associated sepsis, patients with COVID‐19‐associated sepsis, and all patients with sepsis (both entities). The models accurately discriminated between healthy controls and the groups of patients with sepsis, especially in the acute phase of sepsis and to a lesser extent in the late recovery phase of sepsis. The third set of Raman models was specifically targeted to inspect the altered chemical signature of the leukocytes and therein its discriminating power to distinguish between patients with non‐COVID‐19‐ and COVID‐19‐associated sepsis. The discriminatory power of these models was in the fair to substantial range. In the final set of models, we analyzed longitudinal changes (acute phase vs. subacute vs. early and late recovery phase) in Raman spectra. Throughout the different phases, the discriminatory power increased from the time of discharge from the ICU up to 12 months after sepsis onset.

Many epidemiological studies on sepsis have opposing outcomes, which might be related to varying study cohorts, e.g., regarding age and gender [42, 43, 44, 45]. The analysis of Raman spectral data for the influence of age and sex of the patients indicated relatively low discriminative power, except for age in healthy controls, indicating Raman spectroscopy‐based diagnosis being largely unperturbed by patients’ basic demographics. The Raman spectral data obtained from leukocytes predominantly correlate with the sepsis immunopathogenesis, reflecting molecular alterations.

Raman spectral models discriminating between healthy controls and patients with sepsis (non‐COVID‐19‐associated, COVID‐19‐associated, and both) indicate that leukocytes chemically change upon the onset of sepsis from their baseline state, and the alterations are observed until the late recovery phase of sepsis, albeit to a lesser extent. The contributing Raman peaks captured by the PLS‐DA loadings highlight chemical differences such as aminoacids (715 and 814 cm^−1^), lipids (1393 and 2887 cm^−1^), and proteins (1270 and 2935 cm^−1^) (Figure S1). Especially, the leukocytes’ chemical profile in the patients with COVID‐19‐associated sepsis was distinct compared to patients with non‐COVID‐19‐associated sepsis (Table S3). During the late recovery phase, the BAcc/kappa dropped for differentiating patients with sepsis 6 months after sepsis onset and further lowered for 12 months after sepsis onset, indicating leukocytes partially regaining back, their baseline phenotype and functionality. These results are consistent with the previously reported clinical outcomes, which presented the possibility to identify patients with inflammation, infection, and sepsis [34].

The sepsis etiology (non‐COVID‐19‐ vs. COVID‐19‐associated sepsis) was detected with reasonable to significant reliability. Thus, the biochemical profile of the leukocytes differed significantly between both etiologies in the acute phase of sepsis and persisted up to the late recovery phase, albeit with lower discriminative BAcc [46]. The PLS‐DA loadings, featuring the molecular contribution, indicate the specific wavenumber pattern responsible for distinguishing different classes. These patterns change with the underlying cause of sepsis, revealing the significance of these contributing Raman bands and allowing conclusions regarding the responsible class of biomolecules. The Raman spectroscopy data reveal major changes in the amino acids (568 cm^−1^: cysteine, 706 cm^−1^: methionine, 739 cm^−1^: proline, 850 cm^−1^: tryptophan, and 889 cm^−1^: tyrosine) in COVID‐19‐associated sepsis, which are persistent from the acute phase of sepsis to the late recovery phase at 6 months as discerned from the PLS‐DA loadings (Figure S2) [27, 47]. This observation regarding amino acids is consistent with the findings of the study published by Wang et al., which demonstrated upregulation of amino acid metabolism and the group of lipid signaling molecules (eicosanoids) [48, 49]. The difference in the biochemical profile of leukocytes between patients with non‐COVID‐19‐ and COVID‐19‐associated sepsis is observed in overall protein and lipid variations as revealed from the Raman peaks observed in the spectral range (2800–3000 cm^−1^). The difference in the chemical makeup of leukocytes between patients with non‐COVID‐19‐ and COVID‐19‐associated sepsis is insisting post‐sepsis, as visualized from the PLS loading for the late recovery phase 12 months after sepsis onset, shown in Figure S2. These biochemical changes are consistent with previously reported lipidomic and proteomic shifts in septic patients, including decreased high‐density lipoprotein, altered apolipoprotein levels, and disrupted membrane lipid composition. These processes are known to modulate inflammation, immunity, and endothelial integrity [50, 51]. Although the present study did not include direct biochemical validation (e.g., mass spectrometry), the observed spectral differences are consistent with established metabolic and immunological disturbances in sepsis. Future research must integrate complementary methods, such as metabolomics and proteomics, to confirm the precise molecular identities behind the Raman spectral features and further elucidate their relevance to sepsis pathophysiology.

The long‐term effect induced by sepsis on the cellular immune function is an important aspect being addressed by the sepsis research community [21, 35, 52]. Raman spectroscopy allowed longitudinal assessment of leukocytes’ chemical profile in patients with sepsis and monitored the change in the chemical manifestation in patients with non‐COVID‐19‐ and COVID‐19‐associated sepsis. Especially 12 months after sepsis onset (*T*_5_ late recovery phase at 12 months), the models discriminated very well between the acute and recovery phases of sepsis, indicating long‐lasting changes in patients with sepsis (Figures S3 and S4). The contributing molecular component of leukocytes giving rise to respective Raman peaks across the spectrum of PLS‐DA loadings remains consistent at different phases of sepsis (Figure S3), especially for COVID‐19 sepsis (Figure S2), and persists over time. These molecular alterations observed in the leukocytes after a sepsis episode can be extrapolated to the observation made in several clinical observational studies that report reduced quality of life post‐sepsis, with underlying molecular mechanisms involved in immune cell reprogramming [21, 53, 54].

### 4.1 Strengths and Limitations

The strengths of the study are the long period of the follow‐up (12 months) and the assessment of different sepsis entities, namely non‐COVID‐19‐ and COVID‐19‐associated sepsis. Nonetheless, the study has some limitations. The data acquisition for Raman analyses started several months after the initiation of ICROS. This leads to imbalances regarding age and sex in the healthy controls compared to patients with sepsis. Although we found no definitive evidence of systematic bias related to sex or age (see Table S4), and the models’ low accuracy in predicting these demographics suggests minimal influence from these factors, the sepsis detection models exhibited lower model sensitivity in patients with a BMI < 25 kg/m^2^ (see Table S5). In addition, in some patients with sepsis, only blood samples for the late recovery phase were present, reducing the number of patients available for longitudinal comparisons. Moreover, future validation of the proof of principle presented, e.g., regarding Intra‐, Inter‐assay, and overall precision, influence of interfering substances etc., is mandatory on the way to establishing a robust Raman routine method. This study was conducted at a single center, which may limit the generalizability of the findings. Future multicenter studies are warranted to validate these results across diverse clinical settings and patient populations.

## 5 Conclusion

In conclusion, the findings of this study demonstrate the promising diagnostic potential of high‐throughput Raman spectroscopy using leukocytes isolated from minimal blood samples for identifying patients with sepsis. Moreover, this innovative approach opens up the possibility of distinguishing between the underlying causes of sepsis, e.g., non‐COVID‐19‐ versus COVID‐19‐associated sepsis, providing valuable insights for tailored treatment strategies. Notably, the patient's basic demographics (age and sex) showed a relatively low discriminatory power, which indicates the robustness and reliability of this technique across diverse populations. The long‐term assessment of patients with sepsis further reveals persistent biomolecular differences in the leukocytes even after the acute phase of sepsis, shedding light on the lasting alterations in the immune response among patients with sepsis. These findings hold significant implications for the field of sepsis diagnostics and patient management, as Raman spectroscopy shows great potential as a non‐invasive and efficient tool for early detection and monitoring of sepsis. The ability to pinpoint the specific cause of sepsis can aid in more precise and personalized treatment strategies. The study presents a promising perspective of Raman spectroscopy as a label‐free and cost‐effective potential diagnostic platform for identifying changes in the chemical profile of leukocytes throughout the acute and late course of sepsis and to discriminate the etiology of sepsis. In this way, the immune status could be monitored over time to detect an increase or decrease in inflammation and to be able to intervene therapeutically in time.

## Author Contributions

J.P. and S.M.C. contributed to conceptualization. A.R., P.B., and O.R. wrote the original draft. C.N., P.B., and S.M.C. recruited patients, processed blood samples, and supported the clinical analysis. O.R. and T.B. developed machine learning models. A.R., A.P., J.R., and M.K. performed sample preparation, Raman data acquisition, and analysis. O.R., P.B., and T.B. carried out the clinical statistics and modeling. D.V.P. and I.W.S. built the high‐throughput Raman spectrometer and developed the software. S.M.C., C.N., P.B., O.R., A.R., A.S., U.N., J.P., and T.B. supported the interpretation of the clinical assessments. All authors contributed to reviewing and editing the final manuscript.

## Conflicts of Interest

The authors declare no conflicts of interest.

## Supporting information




**Supporting File 1**: biot70105‐sup‐0005‐Appendices.docx.

**Supporting File 2**: biot70105‐sup‐0001‐FigureS1.pptx.

**Supporting File 3**: biot70105‐sup‐0002‐FigureS2.pptx.

**Supporting File 4**: biot70105‐sup‐0003‐FigureS3.pptx.

**Supporting File 5**: biot70105‐sup‐0004‐FigureS4.pptx.

**Supporting File 6**: biot70105‐sup‐0006‐TableS4.xlsx.

**Supporting File 7**: biot70105‐sup‐0007‐TableS5.xlsx:

## Data Availability

The data that support the findings of this study are available from the corresponding author upon reasonable request.

## References

[1] A. Khanina , K. A. Cairns , S. McGloughlin , et al., “Improving Sepsis Care for Hospital Inpatients Using Existing Medical Emergency Response Systems,” Infection, Disease & Health 25, no. 2 (2020): 63–70, 10.1016/j.idh.2019.10.003. 31740379

[2] M. Schinkel , P. W. B. Nanayakkara , and W. J. Wiersinga , “Sepsis Performance Improvement Programs: From Evidence toward Clinical Implementation,” Critical Care 26, no. 1 (2022): 77, 10.1186/s13054-022-03917-1. 35337358 PMC8951662

[3] M. Bauer , H. Gerlach , T. Vogelmann , F. Preissing , J. Stiefel , and D. Adam , “Mortality in Sepsis and Septic Shock in Europe, North America and Australia Between 2009 and 2019— Results From a Systematic Review and Meta‐analysis,” Critical Care (London, England) 24, no. 1 (2020): 239, 10.1186/s13054-020-02950-2. 32430052 PMC7236499

[4] J. Prest , T. Nguyen , T. Rajah , A. B. Prest , M. Sathananthan , and N. Jeganathan , “Sepsis‐Related Mortality Rates and Trends Based on Site of Infection,” Critical Care Explorations 4, no. 10 (2022): 0775, 10.1097/CCE.0000000000000775. PMC9556121 36248320

[5] H. I. Kim and S. Park , “Sepsis: Early Recognition and Optimized Treatment,” Tuberculosis and Respiratory Diseases 82, no. 1 (2019): 6–14, 10.4046/trd.2018.0041. 30302954 PMC6304323

[6] P. E. Marik and J. D. Farkas , “The Changing Paradigm of Sepsis: Early Diagnosis, Early Antibiotics, Early Pressors, and Early Adjuvant Treatment*,” Critical Care Medicine 46, no. 10 (2018): 1690–1692, 10.1097/CCM.0000000000003310. 30216303

[7] K. Leong , B. Gaglani , A. K. Khanna , and M. T. McCurdy , “Novel Diagnostics and Therapeutics in Sepsis,” Biomedicines 9, no. 3 (2021): 311, 10.3390/biomedicines9030311. PMC8003067 33803628

[8] F. Venet and G. Monneret , “Advances in the Understanding and Treatment of Sepsis‐Induced Immunosuppression,” Nature Reviews Nephrology 14, no. 2 (2018): 121–137, 10.1038/nrneph.2017.165. 29225343

[9] T. Barichello , J. S. Generoso , M. Singer , and F. Dal‐Pizzol , “Biomarkers for Sepsis: More Than Just Fever and Leukocytosis—A Narrative Review,” Critical Care (London, England) 26, no. 1 (2022): 14, 10.1186/s13054-021-03862-5. 34991675 PMC8740483

[10] E. Urrechaga , “Reviewing the Value of Leukocytes Cell Population Data (CPD) in the Management of Sepsis,” Annals of Translational Medicine 8, no. 15 (2020): 953, 10.21037/atm-19-3173. 32953753 PMC7475430

[11] M. Singer , C. S. Deutschman , C. W. Seymour , et al., “The Third International Consensus Definitions for Sepsis and Septic Shock (Sepsis‐3),” Journal of the American Medical Association 315, no. 8 (2016): 801–810, 10.1001/jama.2016.0287. 26903338 PMC4968574

[12] J.‐L. Vincent , “The Clinical Challenge of Sepsis Identification and Monitoring,” PLoS Medicine 13, no. 5 (2016): 1002022, 10.1371/journal.pmed.1002022. PMC4871479 27187803

[13] C. F. Duncan , T. Youngstein , M. D. Kirrane , and D. O. Lonsdale , “Diagnostic Challenges in Sepsis,” Current Infectious Disease Reports 23, no. 12 (2021): 22, 10.1007/s11908-021-00765-y. 34720754 PMC8544629

[14] D. M. Needham , J. Davidson , H. Cohen , et al., “Improving Long‐Term Outcomes After Discharge From Intensive Care Unit,” Critical Care Medicine 40, no. 2 (2012): 502–509, 10.1097/CCM.0b013e318232da75. 21946660

[15] A. M. J. Gerth , R. A. Hatch , J. D. Young , and P. J. Watkinson , “Changes in Health‐Related Quality of Life After Discharge From an Intensive Care Unit: A Systematic Review,” Anaesthesia 74, no. 1 (2019): 100–108, 10.1111/anae.14444. PMC6586053 30291744

[16] Y. He , Q. Liu , L. Wei , et al., “The Value of Peripheral Blood Leukocyte Parameters in the Early Diagnosis and Clinical Prognosis of Sepsis,” International Journal of Analytical Chemistry 2023 (2023): 6052085, 10.1155/2023/6052085. 36691469 PMC9867575

[17] N. Bruse , E. J. Kooistra , A. Jansen , et al., “Clinical Sepsis Phenotypes in Critically Ill COVID‐19 Patients,” Critical Care (London, England) 26, no. 1 (2022): 244, 10.1186/s13054-022-04118-6. 35945618 PMC9361232

[18] A. Y. An , A. Baghela , P. Zhang , et al., “Severe COVID‐19 and Non‐COVID‐19 Severe Sepsis Converge Transcriptionally After a Week in the Intensive Care Unit, Indicating Common Disease Mechanisms,” Frontiers in Immunology 14 (2023): 1167917, 10.3389/fimmu.2023.1167917. 37090709 PMC10115984

[19] S. Yende , J. A. Kellum , V. B. Talisa , et al., “Long‐Term Host Immune Response Trajectories among Hospitalized Patients with Sepsis,” JAMA Network Open 2, no. 8 (2019): 198686, 10.1001/jamanetworkopen.2019.8686. PMC6686981 31390038

[20] M. Shankar‐Hari and G. D. Rubenfeld , “Understanding Long‐Term Outcomes Following Sepsis: Implications and Challenges,” Current Infectious Disease Reports 18, no. 11 (2016): 37, 10.1007/s11908-016-0544-7. 27709504 PMC5052282

[21] T. van der Poll , M. Shankar‐Hari , and W. J. Wiersinga , “The Immunology of Sepsis,” Immunity 54, no. 11 (2021): 2450–2464, 10.1016/j.immuni.2021.10.012. 34758337

[22] E. Quansah , E. Gardey , A. Ramoji , et al., “Intestinal Epithelial Barrier Integrity Investigated by Label‐Free Techniques in Ulcerative Colitis Patients,” Scientific Reports 13, no. 1 (2023): 2681, 10.1038/s41598-023-29649-y. 36792686 PMC9931702

[23] A. Scholtz , A. Ramoji , A. Silge , et al., “COVID‐19 Diagnostics: Past, Present, and Future,” ACS Photonics 8, no. 10 (2021): 2827–2838, 10.1021/acsphotonics.1c01052. 37556281

[24] G. P. Birch , T. Campbell , M. Bradley , and K. Dhaliwal , “Optical Molecular Imaging of Inflammatory Cells in Interventional Medicine–An Emerging Strategy,” Frontiers in Oncology 9 (2019): 882, 10.3389/fonc.2019.00882. 31572676 PMC6751259

[25] M. Schwarz , D. Torre , D. Lozano‐Ojalvo , et al., “Rapid, Scalable Assessment of SARS‐CoV‐2 Cellular Immunity by Whole‐Blood PCR,” Nature Biotechnology 40, no. 11 (2022): 1680–1689, 10.1038/s41587-022-01347-6. PMC10603792 35697804

[26] C. E. McCarthy , J. M. White , N. T. Viola , and H. M. Gibson , “In Vivo Imaging Technologies to Monitor the Immune System,” Frontiers in Immunology 11 (2020): 1067, 10.3389/fimmu.2020.01067. 32582173 PMC7280489

[27] H. Salehi , A. Ramoji , S. Mougari , et al., “Specific Intracellular Signature of SARS‐CoV‐2 Infection Using Confocal Raman Microscopy,” Communications Chemistry 5, no. 1 (2022):85, 10.1038/s42004-022-00702-7. PMC9311350 35911504

[28] J. Huang , A. Ramoji , S. Guo , et al., “Vibrational Spectroscopy as a Powerful Tool for Follow‐Up Immunoadsorption Therapy Treatment of Dilated Cardiomyopathy—A Case Report,” Analyst 145, no. 2 (2020): 486–496, 10.1039/c9an01696a. 31781708

[29] J. L. Robertson , R. S. Senger , J. Talty , et al., “Alterations in the Molecular Composition of COVID‐19 Patient Urine, Detected Using Raman Spectroscopic/Computational Analysis,” PLoS ONE 17, no. 7 (2022): 0270914, 10.1371/journal.pone.0270914. PMC9292080 35849572

[30] D. I. Ellis , D. P. Cowcher , L. Ashton , S. O'Hagan , and R. Goodacre , “Illuminating Disease and Enlightening Biomedicine: Raman Spectroscopy as a Diagnostic Tool,” Analyst 138, no. 14 (2013): 3871–3884, 10.1039/c3an00698k. 23722248

[31] J. Lukose , A. K. Barik , N. Mithun , et al., “Raman Spectroscopy for Viral Diagnostics,” Biophysical Reviews 15, no. 2 (2023): 199–221, 10.1007/s12551-023-01059-4. 37113565 PMC10088700

[32] A. Pistiki , F. Hornung , A. Silge , et al., “Raman Spectroscopic Cellomics for the Detection of SARS‐CoV‐2‐Associated Neutrophil Activation After TNF‐α Stimulation,” Clinical and Translational Medicine 12, no. 12 (2022): 1139, 10.1002/ctm2.1139. PMC9763540 36536489

[33] I. W. Schie , J. Rüger , A. S. Mondol , et al., “High‐Throughput Screening Raman Spectroscopy Platform for Label‐Free Cellomics,” Analytical Chemistry 90, no. 3 (2018): 2023–2030, 10.1021/acs.analchem.7b04127. 29286634

[34] A. Ramoji , D. Thomas‐Rüddel , O. Ryabchykov , et al., “Leukocyte Activation Profile Assessed by Raman Spectroscopy Helps Diagnosing Infection and Sepsis,” Critical Care Explorations 3, no. 5 (2021): 0394, 10.1097/CCE.0000000000000394. PMC8162546 34079942

[35] S. M. Coldewey , C. Neu , P. Baumbach , et al., “Identification of Cardiovascular and Molecular Prognostic Factors for the Medium‐Term and Long‐Term Outcomes of Sepsis (ICROS): Protocol for a Prospective Monocentric Cohort Study,” BMJ Open 10, no. 6 (2020): 036527, 10.1136/bmjopen-2019-036527. PMC7312455 32580988

[36] O. Ryabchykov , T. Bocklitz , A. Ramoji , et al., “Automatization of Spike Correction in Raman Spectra of Biological Samples,” Chemometrics and Intelligent Laboratory Systems 155 (2016): 1–6, 10.1016/j.chemolab.2016.03.024.

[37] S. Guo , R. Heinke , S. Stöckel , P. Rösch , J. Popp , and T. Bocklitz , “Model Transfer for Raman‐Spectroscopy‐Based Bacterial Classification,” Journal of Raman Specroscopy 49, no. 4 (2018): 627–637, 10.1002/jrs.5343. 30016081

[38] U. G. Indahl , K. H. Liland , and T. Naes , “Canonical Partial Least Squares—A Unified PLS Approach to Classification and Regression Problems,” Journal of Chemometrics 23, no. 9 (2009): 495–504, 10.1002/cem.1243.

[39] B.‐H. Mevik and R. Wehrens , “The Pls Package: Principal Component and Partial Least Squares Regression in R,” Journal of Statistical Software 18, no. 2 (2007): 1–23, 10.18637/jss.v018.i02.

[40] M. L. McHugh , “Interrater Reliability: The kappa Statistic,” Biochemia Medica 22, no. 3 (2012): 276–282. 23092060 PMC3900052

[41] N. Ali , S. Girnus , P. Rösch , J. Popp , and T. Bocklitz , “Sample‐Size Planning for Multivariate Data: A Raman‐Spectroscopy‐Based Example,” Analytical Chemistry 90, no. 21 (2018): 12485–12492, 10.1021/acs.analchem.8b02167. 30272961

[42] R.‐E. Ko , D. Kang , J. Cho , et al., “Influence of Gender on Age‐Associated In‐Hospital Mortality in Patients With Sepsis and Septic Shock: A Prospective Nationwide Multicenter Cohort Study,” Critical Care (London, England) 27, no. 1 (2023): 229, 10.1186/s13054-023-04515-5. 37303037 PMC10257805

[43] S. R. Eachempati , L. Hydo , and P. S. Barie , “Gender‐Based Differences in Outcome in Patients With Sepsis,” Archives of Surgery (Chicago, Ill: 1960) 134, no. 12 (1999): 1342–1347, 10.1001/archsurg.134.12.1342. 10593332

[44] K. Thompson , N. Hammond , M. Bailey , et al., “Sex Differences in Long‐Term Survival After Intensive Care Unit Treatment for Sepsis: A Cohort Study,” PLoS ONE 18, no. 2 (2023): e0281939, 10.1371/journal.pone.0281939. PMC9955961 36827250

[45] K. J. Thompson , S. R. Finfer , M. Woodward , R. N. F. Leong , and B. Liu , “Sex Differences in Sepsis Hospitalisations and Outcomes in Older Women and Men: A Prospective Cohort Study,” Journal of Infection 84, no. 6 (2022): 770–776, 10.1016/j.jinf.2022.04.035. 35472366

[46] A. Herminghaus and M. F. Osuchowski , “How Sepsis Parallels and Differs From COVID‐19,” EBioMedicine 86 (2022): 104355, 10.1016/j.ebiom.2022.104355. 36470836 PMC9718536

[47] R. Maeda , N. Seki , Y. Uwamino , et al., “Amino Acid Catabolite Markers for Early Prognostication of Pneumonia in Patients With COVID‐19,” Nature Communications 14, no. 1 (2023): 8469, 10.1038/s41467-023-44266-z. PMC10733290 38123556

[48] J. Wang , Y. Sun , S. Teng , and K. Li , “Prediction of Sepsis Mortality Using Metabolite Biomarkers in the Blood: A Meta‐Analysis of Death‐Related Pathways and Prospective Validation,” BMC Medicine 18, no. 1 (2020): 83, 10.1186/s12916-020-01546-5. 32290837 PMC7157979

[49] K. Amunugama , D. P. Pike , and D. A. Ford , “The Lipid Biology of Sepsis,” Journal of Lipid Research 62 (2021): 100090, 10.1016/j.jlr.2021.100090. 34087197 PMC8243525

[50] R. J. Langley , E. L. Tsalik , J. C. van Velkinburgh , et al., “An Integrated Clinico‐Metabolomic Model Improves Prediction of Death in Sepsis,” Science Translational Medicine 5, no. 195 (2013): 195ra95, 10.1126/scitranslmed.3005893. PMC3924586 23884467

[51] N. K. Sharma , B. L. Ferreira , A. K. Tashima , et al., “Lipid Metabolism Impairment in Patients With Sepsis Secondary to Hospital Acquired Pneumonia, a Proteomic Analysis,” Clinical Proteomics 16 (2019): 29, 10.1186/s12014-019-9252-2. 31341447 PMC6631513

[52] M. J. Delano and P. A. Ward , “The Immune System's Role in Sepsis Progression, Resolution, and Long‐Term Outcome,” Immunological Reviews 274, no. 1 (2016): 330–353, 10.1111/imr.12499. 27782333 PMC5111634

[53] H. C. Prescott , J. B. Sussman , and W. J. Wiersinga , “Postcritical Illness Vulnerability,” Current Opinion in Critical Care 26, no. 5 (2020): 500–507, 10.1097/MCC.0000000000000761. 32773618

[54] A. Roquilly , C. Jacqueline , M. Davieau , et al., “Alveolar Macrophages Are Epigenetically Altered After Inflammation, Leading to Long‐Term Lung Immunoparalysis,” Nature Immunology 21, no. 6 (2020): 636–648, 10.1038/s41590-020-0673-x. 32424365

