# Supplementary material for: Raman Spectroscopy Can Identify Acute and Persistent Biochemical Changes in Leukocytes From Patients With COVID‐19 and Non‐COVID‐19‐Associated Sepsis
Source: Biotechnol J. 2025 Sep 1;20(9):e70105. doi: 10.1002/biot.70105 (PMC12402750; doi:10.1002/biot.70105)
Supplement: Supplementary file 1 — Supporting File 1: biot70105‐sup‐0005‐Appendices.docx. [file BIOT-20-e70105-s007.docx]

**Raman spectroscopy can identify acute and persistent biochemical changes in leukocytes from patients with COVID-19 and non-COVID-19-associated sepsis**

**Running title:** Raman spectroscopy in non- and COVID-19-associated sepsis

*Anuradha Ramoji^a,b,†^, Philipp Baumbach^c,d,†^, Oleg Ryabchykov^a,b,†^, Aikaterini Pistiki^a,b^, Jan Rueger^b^, David Vasquez Pinzon^b^, Anja Silge^b^, Stefanie Deinhardt-Emmer^e^, Iwan W. Schie^b,f^, Karina Weber^b^, Charles Neu^c,d^, Ute Neugebauer^a,b,g^, Michael Kiehntopf^h^, Thomas Bocklitz^a,b,*^, Juergen Popp^a,b,*^, Sina M. Coldewey^i,c,d,g,*^*

^a^ Friedrich Schiller University Jena, Institute of Physical Chemistry and Abbe Center of Photonics, Helmholtzweg 4, 07743 Jena, Germany, member of the Leibniz Centre for Photonics in Infection Research (LPI), Jena, Germany

^b^ Leibniz Institute of Photonic Technology - Member of the research alliance “Leibniz Health Technologies “, Albert-Einstein-Straße 9, 07745 Jena, Germany, member of the Leibniz Centre for Photonics in Infection Research (LPI), Jena, Germany

^c^ Jena University Hospital, Department of Anesthesiology and Intensive Care, Friedrich-Schiller-University Jena, Am Klinikum 1, 07747, Jena, Germany.

^d^ Friedrich-Schiller-University Jena, Septomics Research Centre, Jena University Hospital, Albert-Einstein-Straße 10, 07745 Jena, Germany.

^e^ Jena University Hospital, Institute of Medical Microbiology, Friedrich-Schiller-University Jena, Am Klinikum 1, 07747 Jena, Germany

^f^ University of Applied Sciences Jena, Department of Medical Engineering and Biotechnology, Carl-Zeiss-Promenade 2, 07745 Jena, Germany

^g^ Jena University Hospital, Center for Sepsis Control and Care, Friedrich-Schiller-University Jena, Am Klinikum 1, 07747 Jena, Germany.

^h^ Jena University Hospital, Institute for Clinical Chemistry and Laboratory Diagnostics, Am Klinikum 1, 07747 Jena, Germany

^i^ Institute of Anesthesiology and Perioperative Medicine, University Hospital Zurich, University of Zurich, Raemistrasse 100, 8091 Zurich, Switzerland

^†^ Anuradha Ramoji, Philipp Baumbach, and Oleg Ryabchykov contributed equally to this work.

***** Correspondence: Sina Coldewey (clinical trial, study design), [sina.coldewey@uzh.ch](mailto:sina.coldewey@uzh.ch), Juergen Popp (Raman spectroscopy), [juergen.popp@Leibniz-ipht.de](mailto:juergen.popp@Leibniz-ipht.de), Thomas Bocklitz (Data Science), [thomas.bocklitz@uni-jena.de](mailto:thomas.bocklitz@uni-jena.de)

**APPENDIX A**

**Sample preparation and Raman measurements**

Blood sampling

Blood samples have been collected following standard protocols, blood (2.7 ml) was collected into Ethylenediaminetetraacetic acid (EDTA) containing tubes (Sarstedt, Nuembrecht, Germany). For isolation of the leukocytes, 500µl of blood was used and the remaining blood was transferred to routine clinical analysis.

Leukocytes isolation

The blood collection protocols and leukocyte isolation procedure have been published previously [1] and are summarized here briefly. EDTA blood of 500µl volume was transferred into a falcon tube for the isolation of the leukocytes. Leukocytes were isolated by removing erythrocytes (RBC) by cytolysis method using BD FACS lysing solution (BD Bioscience, CA, USA)). The BD FACS lysing solution contains a formaldehyde-based fixative solution that lyses the RBCs and chemically fixes the cells. Afterward, the cells were washed with PBS. The leukocytes were stored at 4^o^C and investigated within 48 h after preparation.

Raman spectroscopy measurements

Raman spectra of the leukocytes were recorded using high throughput (HT) Raman device which is faster than lab-device, 1000 cells within 30 mins can be measured with 1s of laser exposure per cell. The HT Raman system is an upright Raman microscope equipped with a 785 nm single-mode excitation laser with a power of 100 mW at the plane of the objective. The spectrometer is equipped with a grating of 400 grooves/mm, blazed at 750 nm, and allows a spectral resolution of 9 cm^-1^. For the bright field image acquisition, the sample can be illuminated from the bottom using a standard white light LED and detected on the CCD camera. The Raman module has an inbuilt software developed using the LabView platform which allows automatic selection of cells and acquisition of Raman spectra via 60x/1.0 NA water immersion Nikon objective. The software allows capturing bright-field images of the cells by moving the motorized x-y translation stage. A custom-made algorithm [2] allowed the automatic selection of the cells followed by the acquisition of the Raman spectra. The isolated leukocytes were re-suspended in PBS with a cell concentration of 4 x 106/ml and ~20μl of cell suspension was placed on the calcium fluoride slide in a petri dish filled with distilled water. The cells were allowed to settle down for a few minutes (~5 min). Bright-field images of the cells were captured, and the Raman spectrum was recorded. For each patient, approximately 1500 cells were measured.

Analysis of Raman spectra

Raman spectra were pre-processed to minimize sample-to-sample variations, which is a prerequisite for the utilization of the tiny spectral changes between the Raman spectra of the disease states [3]. First, the spectra were corrected for cosmic ray noise, then they were calibrated according to the measured standard spectra of 4-acetamidophenol. Furthermore, noisy spectra were excluded based on the Euclidean distance between the SNIP baseline-corrected spectra and the reference spectrum. The threshold for spectra exclusion was set to four times the median distance from the reference spectrum over the whole data set. After selecting the spectra of good quality, they were standardized using a model-based pre-processing method called extended multiplicative scatter correction (EMSC). The advantage of the method is that the spectra do not require further normalization and it allows standardizing spectra obtained from different sources using the same reference. In the below **Figure A1** raw and preprocessed Raman spectra of healthy and patients with sepsis have been displayed. Further analysis of the preprocessed spectra was performed by partial least-squares discriminant analysis (PLS-DA) regression analysis.

| 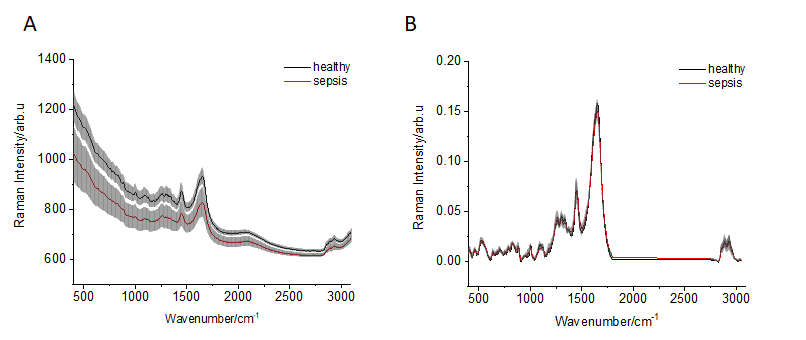 |
| --- |
| **Figure A1** A) Raw and B) preprocessed Raman spectra shown for healthy and patients with sepsis |

The number of PLS latent variables (LV) was optimized in a patient-out cross validation with the maximum of 20 LV. The high-performing models are robust over small changes in the number of LV (**Figure A2 A-C**), which suggests that the model performance is not overestimated. Furthermore, overfitting would lead to a decrease in the model performance, which is apparent in **Figure A2 D**.

| 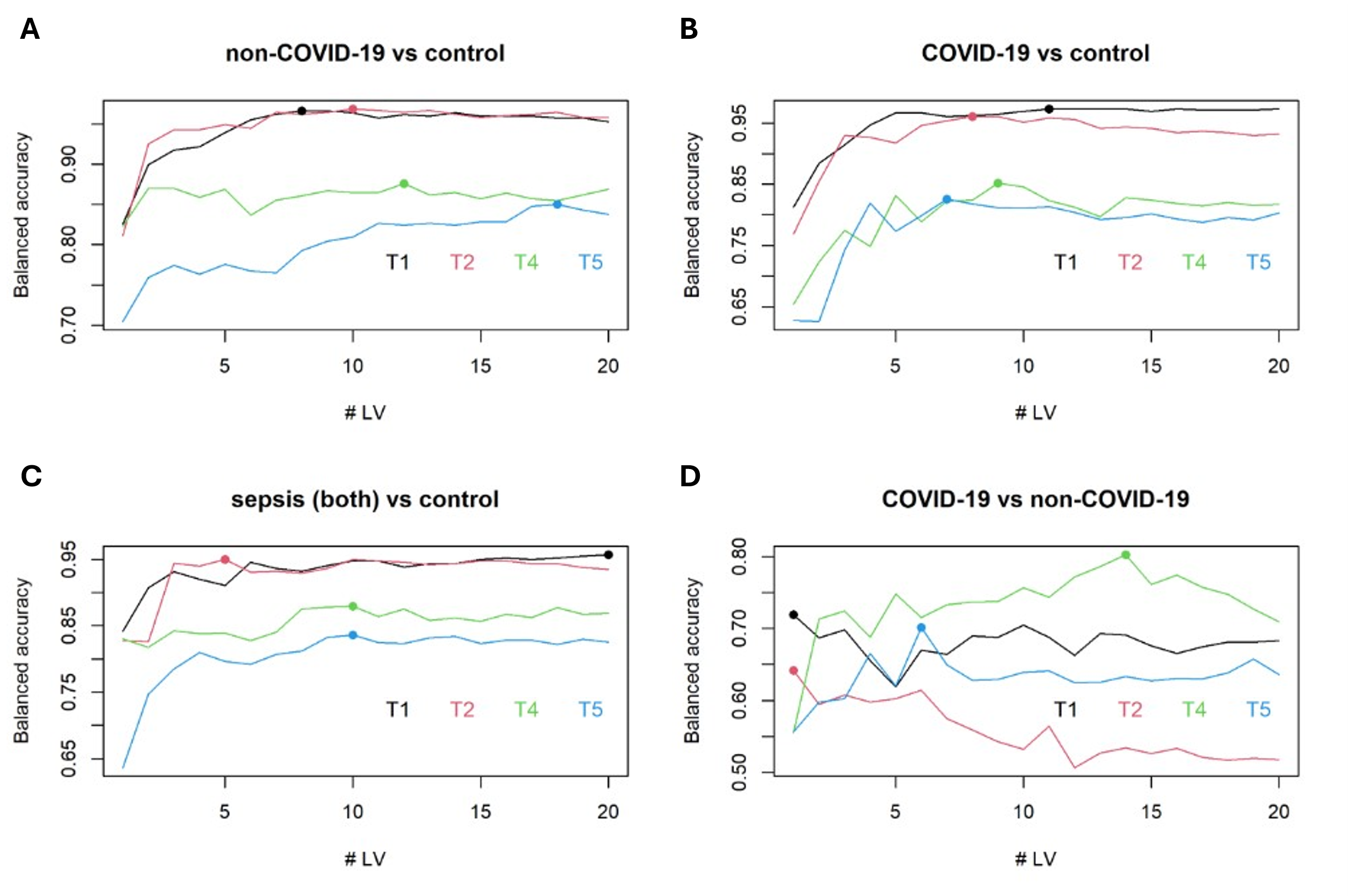 |
| --- |
| **Figure A2** Cross-validation performance with varying number of partial least-squares latent variables (LV). Due to cross-validation on the patient level, the overfitting does not result in increase of accuracy, featuring either a “saturation” or even a decrease of the model performance with increased number of LV. |

**Supplementary APPENDIX B**

**TABLE S1** Overview of patient numbers for the different Raman spectral-based models (T_1_: 3±1 days after sepsis onset, T_2_: 7±1 days after sepsis onset, T_3_: ± 3 days after discharge from intensive care unit, T_4_: 6±2 months after sepsis onset, T_5_: 12±2 months after sepsis onset).

| **Sex** | **n_female_** | **n_male_** |
| --- | --- | --- |
| Healthy | 7 | 37 |
| Sepsis: all (T_1_, 3±1 days after sepsis onset) | 30 | 64 |
| Sepsis: non-COVID-19 (T_1_, 3±1 days after sepsis onset) | 16 | 29 |
| Sepsis: COVID-19 (T_1_, 3±1 days after sepsis onset) | 14 | 35 |
| **Age** | **n_<65 years_** | **n_≥65 years_** |
| Healthy | 22 | 22 |
| Sepsis: all (T_1_, 3±1 days after sepsis onset) | 47 | 47 |
| Sepsis: non-COVID-19 (T_1_, 3±1 days after sepsis onset) | 23 | 22 |
| Sepsis: COVID-19 (T_1_, 3±1 days after sepsis onset) | 24 | 25 |
| **Sepsis *vs* healthy controls** | **n_Sepsis_** | **n_Healthy_** |
| **Acute phase of sepsis** |  |  |
| T_1_ (3±1 days after sepsis onset) |  |  |
| *Sepsis: all* | 94 | 44 |
| *Sepsis: non-COVID-19* | 45 | 44 |
| *Sepsis: COVID-19* | 49 | 44 |
| T_2_ (7±1 days after sepsis onset) |  |  |
| *Sepsis: all* | 88 | 44 |
| *Sepsis: non-COVID-19* | 42 | 44 |
| *Sepsis: COVID-19* | 46 | 44 |
| **Late recovery phase of sepsis** |  |  |
| T_4_ (6±2 months after sepsis onset) |  |  |
| *Sepsis: all* | 75 | 44 |
| *Sepsis: non-COVID-19* | 42 | 44 |
| *Sepsis: COVID-19* | 33 | 44 |
| T_5_ (12±2 months after sepsis onset) |  |  |
| *Sepsis: all* | 73 | 44 |
| *Sepsis: non-COVID-19* | 40 | 44 |
| *Sepsis: COVID-19* | 33 | 44 |
| **Sepsis: non-COVID-19 *vs* COVID-19** | **n_non-COVID-19_** | **n_COVID-19_** |
| **Acute phase of sepsis** |  |  |
| T_1_ (3±1 days after sepsis onset) | 45 | 49 |
| T_2_ (7±1 days after sepsis onset) | 42 | 46 |
| **Late recovery phase of sepsis** |  |  |
| T_4_ (6±2 months after sepsis onset) | 42 | 33 |
| T_5_ (12±2 months after sepsis onset) | 40 | 33 |
| **Longitudinal changes** | **n_Sepsis_** |  |
| **Early recovery: T_1_ *vs* T_3_** |  |  |
| *Sepsis: all* | 61 |  |
| *Sepsis: non-COVID-19* | 32 |  |
| *Sepsis: COVID-19* | 29 |  |
| **Late recovery: T_1_ *vs* T_4_** |  |  |
| *Sepsis: all* | 52 |  |
| *Sepsis: non-COVID-19* | 20 |  |
| *Sepsis: COVID-19* | 32 |  |
| **Late recovery: T_1_ *vs* T_5_** |  |  |
| *Sepsis: all* | 47 |  |
| *Sepsis: non-COVID-19* | 15 |  |
| *Sepsis: COVID-19* | 32 |  |

**TABLE S2** Sensitivity obtained using partial least-squares discriminant analysis for the reported Raman spectral-based models for investigating the influence of patients’ age and sex.

| **Raman Models** | **n_Class 1_** | **n_Class 2_** | **Sensitivity** | **Specificity** | **Balanced**  **accuracy** | **Cohen’s**  ***kappa*** |
| --- | --- | --- | --- | --- | --- | --- |
| **Age (Class 1: < 65 *vs* Class 2: ≥ 65 years)** |  |  |  |  |  |  |
| Healthy | 22 | 22 | 0.77 | 0.77 | 0.77 | 0.55 |
| Non-COVID-19-associated sepsis | 23 | 22 | 0.48 | 0.68 | 0.58 | 0.16 |
| COVID-19-associated sepsis | 24 | 25 | 0.54 | 0.72 | 0.63 | 0.29 |
| Sepsis (both) | 47 | 47 | 0.57 | 0.60 | 0.59 | 0.26 |
| **Sex (Class 1: female *vs* Classe 2: male)** |  |  |  |  |  |  |
| Healthy | 7 | 37 | 0.14 | 1.00 | 0.57 | 0.22 |
| Non-COVID-19-associated sepsis | 16 | 29 | 0.25 | 0.79 | 0.52 | 0.05 |
| COVID-19-associated sepsis | 14 | 35 | 0.07 | 0.94 | 0.51 | 0.02 |
| Sepsis (both) | 30 | 64 | 0.23 | 0.86 | 0.55 | 0.11 |

**TABLE S3** Discriminatory power of the reported Raman spectral-based models using partial least-squares discriminant analysis.

| **Raman Models** | **n_Class1_** | **n_Class2_** | **Sensitivity** | **Specificity** | **Balanced**  **accuracy** | **Cohen’s kappa** |
| --- | --- | --- | --- | --- | --- | --- |
| **Patients with sepsis *vs* healthy controls (Class 2)** |  |  |  |  |  |  |
| **Acute phase of sepsis** |  |  |  |  |  |  |
| **T_1_ (3±1 days after sepsis onset)** |  |  |  |  |  |  |
| Non-COVID-19-associated sepsis (Class 1) | 45 | 44 | 0.96 | 0.98 | 0.97 | 0.93 |
| COVID-19-associated sepsis (Class 1) | 49 | 44 | 1.00 | 0.95 | 0.98 | 0.96 |
| Sepsis (both, Class 1) | 94 | 44 | 1.00 | 0.95 | 0.98 | 0.97 |
| **T_2_ (7±1 days after sepsis onset)** |  |  |  |  |  |  |
| Non-COVID-19-associated sepsis (Class 1) | 42 | 44 | 0.98 | 0.95 | 0.97 | 0.93 |
| COVID-19-associated sepsis (Class 1) | 46 | 44 | 0.98 | 0.95 | 0.97 | 0.93 |
| Sepsis (both, Class 1) | 88 | 44 | 1.00 | 0.91 | 0.95 | 0.93 |
| **Subacute phase of sepsis *vs* healthy control (Class 2)** |  |  |  |  |  |  |
| **T3 (±3 days after ICU-discharge)** |  |  |  |  |  |  |
| Non-COVID-19-associated sepsis (Class 1) | 33 | 44 | 0.91 | 0.98 | 0.94 | 0.89 |
| COVID-19-associated sepsis (Class 1) | 21 | 44 | 0.71 | 0.98 | 0.85 | 0.74 |
| Sepsis (both, Class 1) | 54 | 44 | 0.98 | 0.95 | 0.97 | 0.94 |
| **Late recovery phase of sepsis *vs* healthy control** |  |  |  |  |  |  |
| **T_4_ (6±2 months after sepsis onset)** |  |  |  |  |  |  |
| Non-COVID-19-associated sepsis (Class 1) | 42 | 44 | 0.88 | 0.89 | 0.88 | 0.77 |
| COVID-19-associated sepsis (Class 1) | 33 | 44 | 0.79 | 0.91 | 0.85 | 0.70 |
| Sepsis (both, Class 1) | 75 | 44 | 0.95 | 0.82 | 0.88 | 0.78 |
| **T_5_ (12±2 months after sepsis onset)** |  |  |  |  |  |  |
| Non-COVID-19-associated sepsis (Class 1) | 40 | 44 | 0.88 | 0.93 | 0.90 | 0.81 |
| COVID-19-associated sepsis (Class 1) | 33 | 44 | 0.79 | 0.84 | 0.81 | 0.63 |
| Sepsis (both, Class 1) | 73 | 44 | 0.95 | 0.73 | 0.84 | 0.70 |
| **COVID-19- (Class 1) *vs* non-COVID-19-associated sepsis (Class 2)** | | | | | | |
| **Acute phase of sepsis** |  |  |  |  |  |  |
| T_1_ (3±1 days after sepsis onset) | 49 | 45 | 0.71 | 0.71 | 0.71 | 0.43 |
| T_2_ (7±1 days after sepsis onset) | 46 | 42 | 0.78 | 0.52 | 0.65 | 0.31 |
| **Subacute phase of sepsis** |  |  |  |  |  |  |
| T_3_ (±3 days after ICU-discharge) | 21 | 33 | 0.57 | 0.82 | 0.69 | 0.40 |
| **Late recovery phase of sepsis** |  |  |  |  |  |  |
| T_4_ (6±2 months after sepsis onset) | 33 | 42 | 0.82 | 0.83 | 0.83 | 0.65 |
| T_5_ (12±2 months after sepsis onset) | 33 | 40 | 0.67 | 0.75 | 0.71 | 0.42 |
| **Longitudinal changes *vs* T_1_ (3±1 days after sepsis onset)** |  |  |  |  |  |  |
| **Subacute phase: T_3_ (discharge from ICU) *vs* T_1_** |  |  |  |  |  |  |
| Non-COVID-19-associated sepsis (T_3_ *vs* T_1_) | 32 | 32 | 0.59 | 0.59 | 0.59 | 0.19 |
| COVID-19-associated sepsis (T_3_ *vs* T_1_) | 29 | 29 | 0.62 | 0.59 | 0.60 | 0.21 |
| Sepsis (T_3_ *vs* T_1_) | 61 | 61 | 0.61 | 0.54 | 0.57 | 0.15 |
| **Late recovery phase: T_4_ (6±2 months after sepsis onset) *vs* T_1_** |  |  |  |  |  |  |
| Non-COVID-19-associated sepsis (T_4_ *vs* T_1_) | 20 | 20 | 0.95 | 0.85 | 0.90 | 0.80 |
| COVID-19-associated sepsis (T_4_ *vs* T_1_) | 32 | 32 | 0.81 | 0.88 | 0.84 | 0.69 |
| Sepsis (T_4_ *vs* T_1_) | 52 | 52 | 0.85 | 0.87 | 0.86 | 0.71 |
| **Late recovery phase: T_5_ (12±2 months after sepsis onset) *vs* T_1_** |  |  |  |  |  |  |
| Non-COVID-19-associated sepsis (T_5_ *vs* T_1_) | 15 | 15 | 0.87 | 0.87 | 0.87 | 0.73 |
| COVID-19-associated sepsis (T_5_ *vs* T_1_) | 32 | 32 | 0.97 | 0.97 | 0.97 | 0.94 |
| Sepsis (T_5_ *vs* T_1_) | 47 | 47 | 0.98 | 0.96 | 0.97 | 0.94 |

| 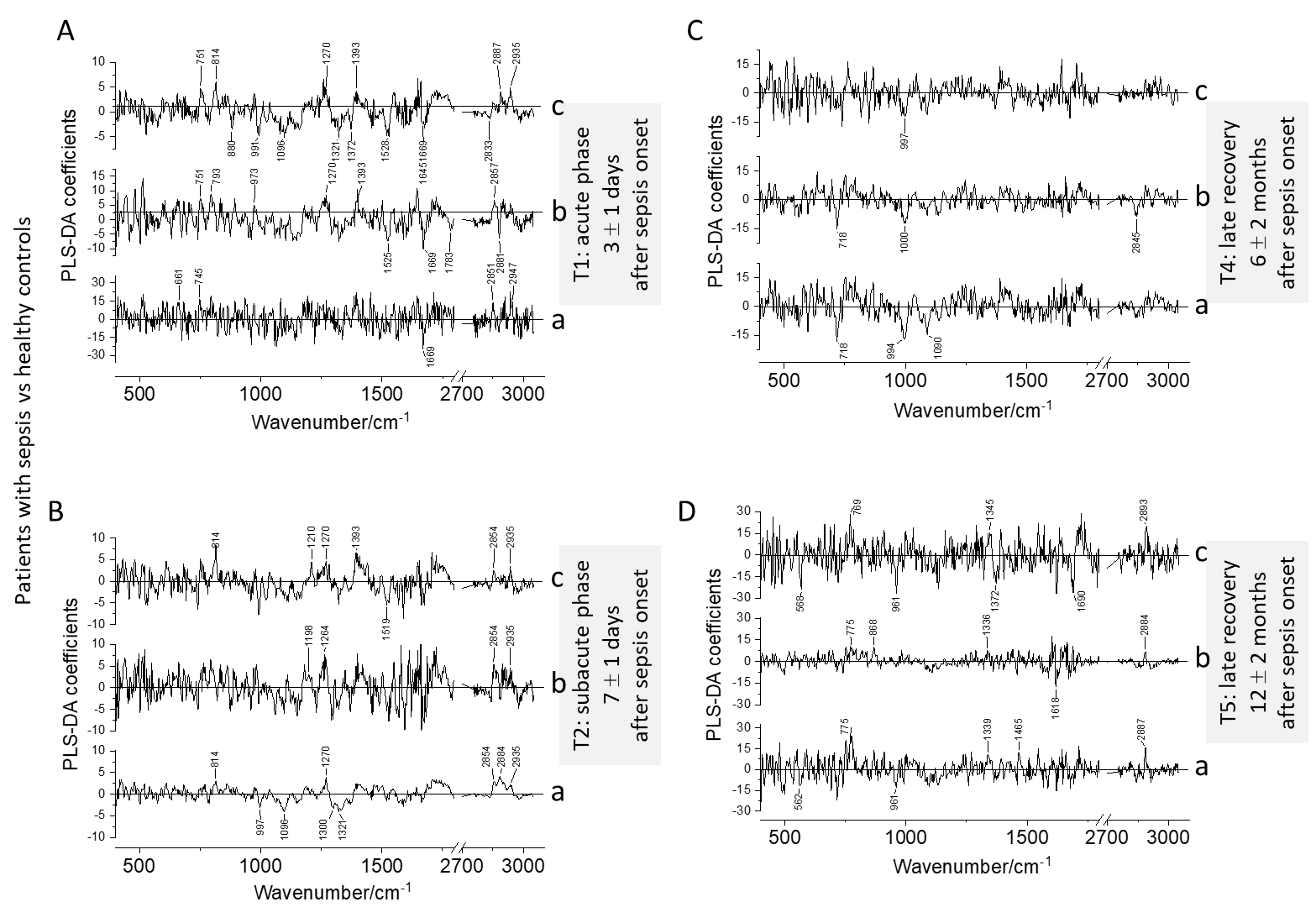 |
| --- |
| **FIGURE S1** Loading coefficients showing Raman bands of peripheral leukocytes contributing to discriminating healthy controls and patients with sepsis (model performance shown in Figure 3, main manuscript). The loadings coefficients obtained from the Raman model for the **A)** acute phase (T_1_: 3±1 days), **B)** subacute phase (T_2_: 7±1 days after sepsis onset), **C and D)** late recovery phases (T_4_: 6±2 months and T_5_: 12±2 months after sepsis onset). a: sepsis (both), b: COVID-19-associated sepsis, c: non-COVID-19-associated sepsis. Positive Raman bands are prominent in sepsis patients and negative Raman bands are prominent in the healthy controls. |

| 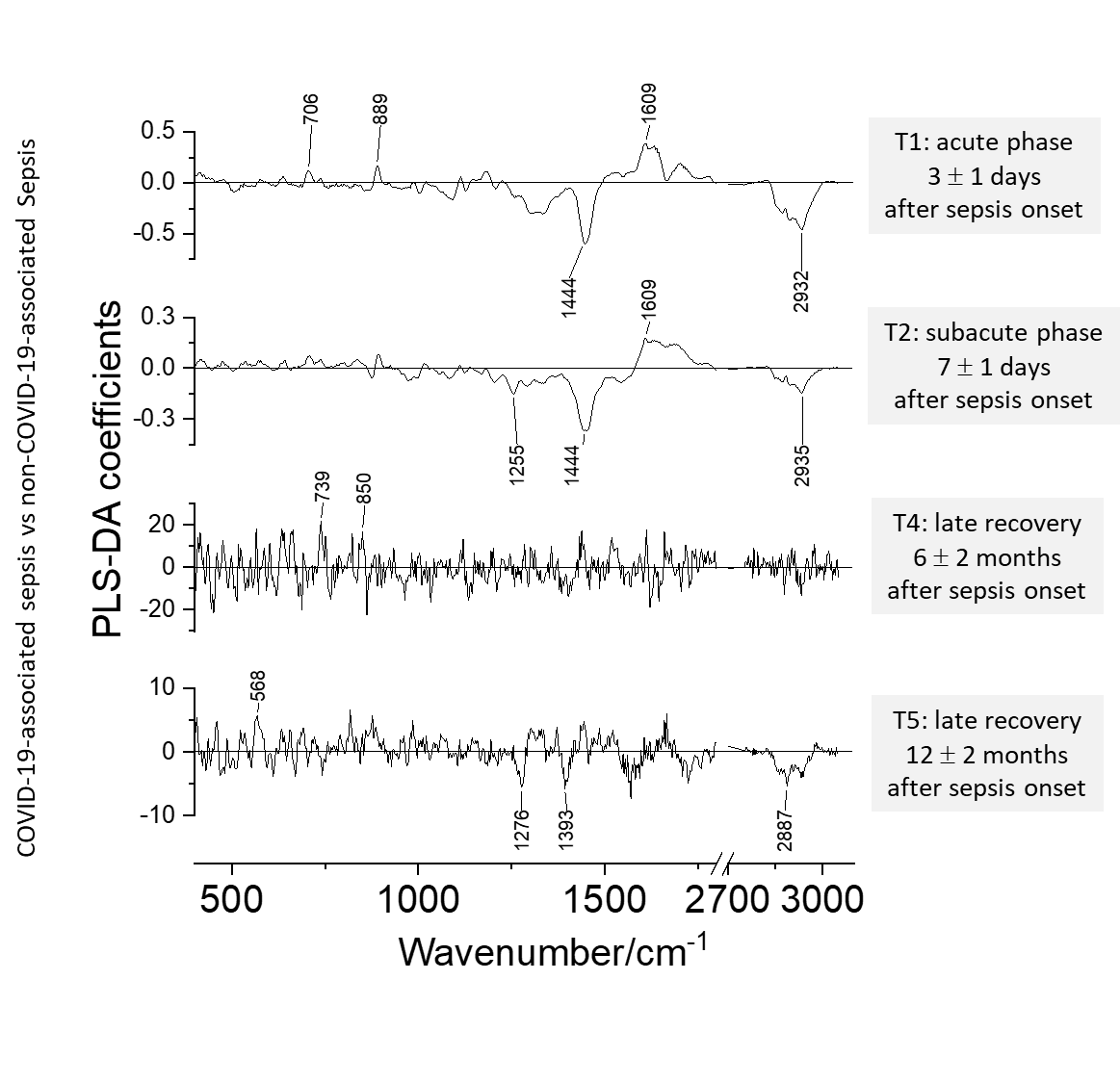 |
| --- |
| **FIGURE S2** Loading coefficients showing Raman bands of peripheral leukocytes contributing to discriminating between patients with non-COVID-19- and COVID-19-associated sepsis (model performance shown in Figure 4, main manuscript). The loadings coefficients obtained from the Raman model for the acute phase (T_1_: 3±1 days after sepsis onset), subacute phase (T_2_: 7±1 days after sepsis onset), and late recovery phases (T_4_: 6±2 months and T_5_: 12±2 months after sepsis onset). Positive Raman bands are prominent in COVID-19-associated sepsis patients and negative Raman bands are prominent in the patients with non-COVID-19-associated sepsis. |

| 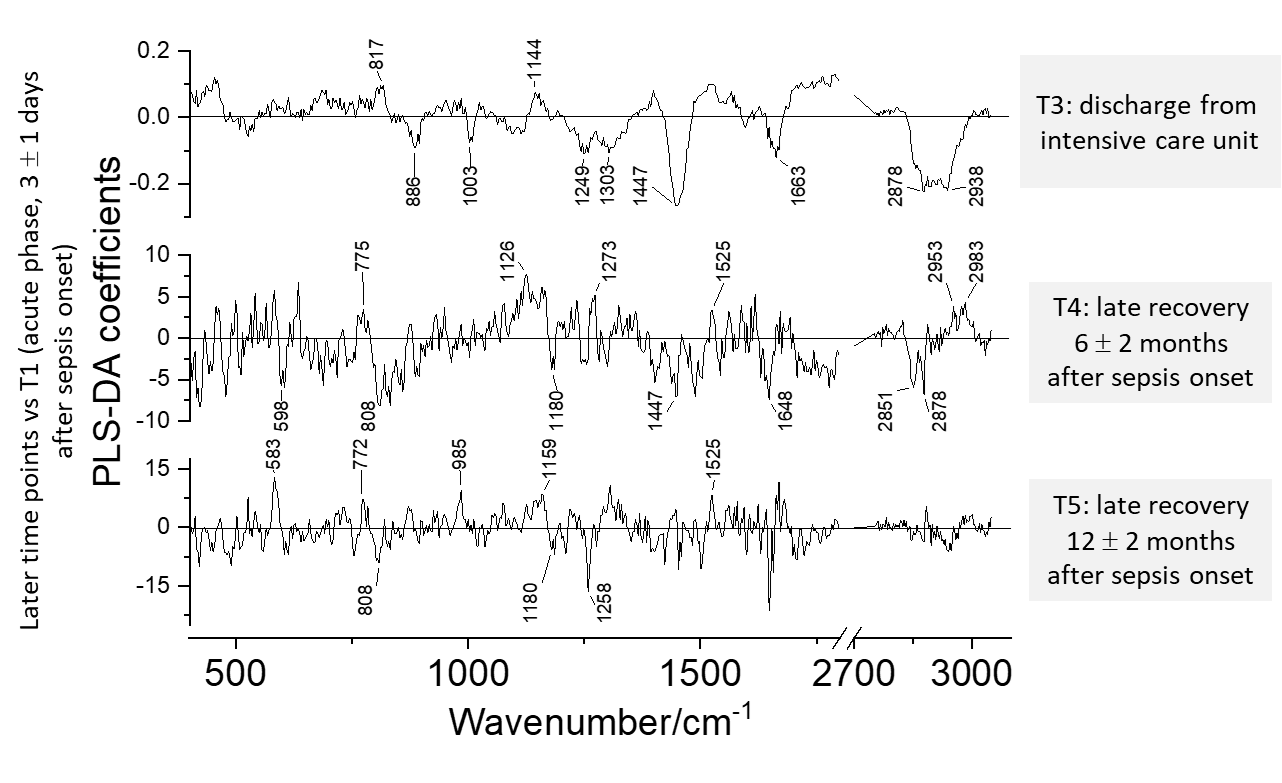 |
| --- |
| **FIGURE S3** Loading coefficients showing Raman bands of peripheral leukocytes contributing to the longitudinal changes in cells of patients with sepsis (patients with non-COVID-19- and COVID-19-associated sepsis). Results are shown for early (discharge from intensive care unit) and late recovery phases (model performance shown in Figure 5, main manuscript). Positive Raman bands are prominent in early (T_3_: discharge from intensive care unit) and later recovery (T_4_: 6±2 months and T_5_: 12±2 months after sepsis onset). and negative Raman bands are prominent in the acute phase of sepsis (T_1_: 3 ± 1 days after sepsis onset). |

| 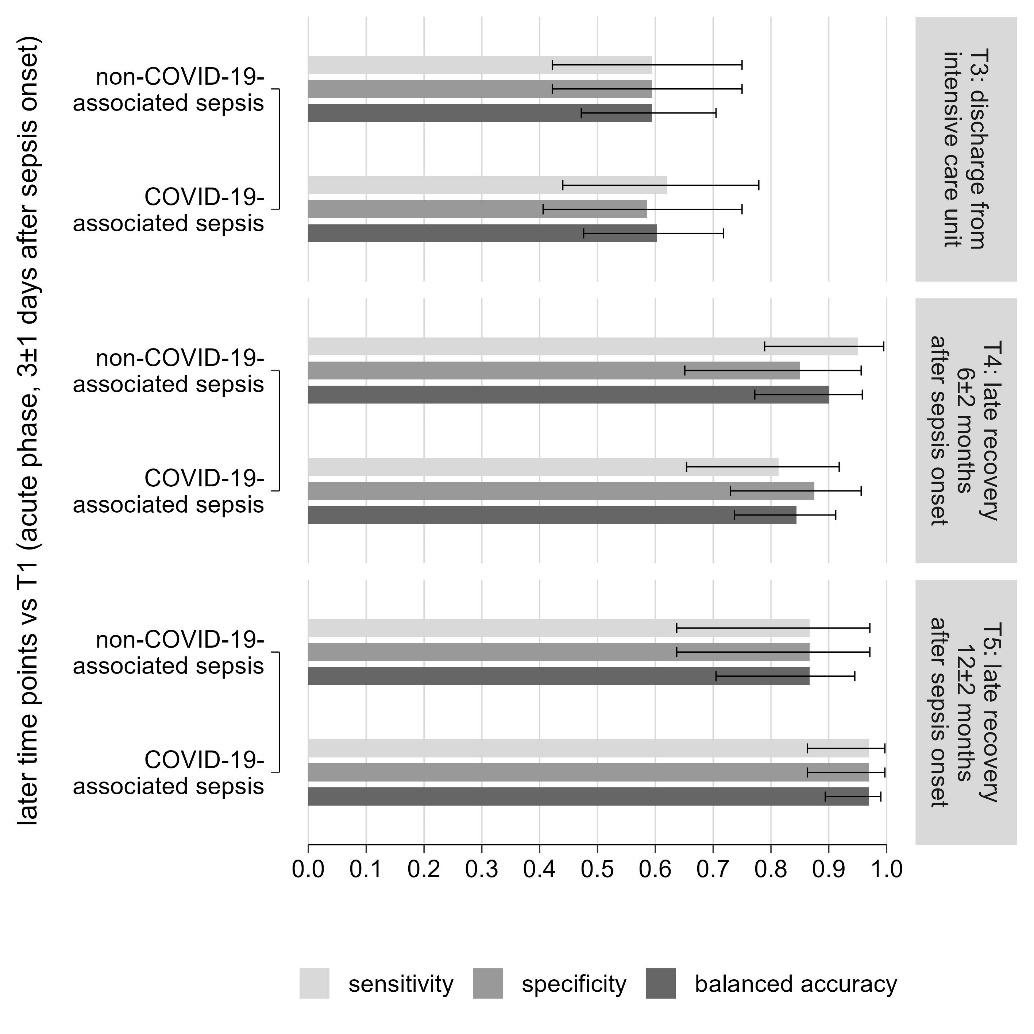 |
| --- |
| **FIGURE S4** Raman spectroscopy investigations of longitudinal changes in peripheral leukocytes from patients with non-COVID-19- and COVID-19-associated sepsis. The bar graph shows sensitivity, specificity, and balanced accuracy (including 95% confidence intervals) obtained from Raman spectral-based models using partial least-squares discriminant analysis for detecting longitudinal changes. Results are presented for discharge from the intensive care unit and the late recovery phase. |

**TABLE S4** Stability of the models over four demographic groups based on age and sex. The color fill highlights low values for visualization purposes. Sensitivity (Sens) and specificity (Spec) values remain in balance among and across most demographic groups for majority of tasks. The exception is the adult female group, which has an obvious discrepancy with 0% sensitivity, but it should be noted that this value is calculated based on a single volunteer and is not sufficient for drawing. The overall uniformity of the results suggests that there is no significant demographic bias in the models.

|  | | | Adult (18-64 years) | | | | Geriatric (65+ years) | | | |
| --- | --- | --- | --- | --- | --- | --- | --- | --- | --- | --- |
|  |  |  | male | | female | | male | | female | |
| **Task / Sepsis type** | | **Phase** | **Sens** | **Spec** | **Sens** | **Spec** | **Sens** | **Spec** | **Sens** | **Spec** |
| patients with sepsis *vs* healthy | non-COVID-19 | T1: acute | 1.00 | 0.95 | 0.86 | 1.00* | 1.00 | 1.00 | 0.89 | 1.00 |
|  | COVID-19 |  | 1.00 | 0.95 | 1.00 | 0.00* | 1.00 | 1.00 | 1.00 | 1.00 |
|  | both |  | 1.00 | 0.95 | 1.00 | 0.00* | 1.00 | 1.00 | 1.00 | 1.00 |
|  | non-COVID-19 | T2: subacute | 1.00 | 0.95 | 1.00 | 0.00* | 0.91 | 1.00 | 1.00 | 1.00 |
|  | COVID-19 |  | 0.94 | 0.95 | 1.00 | 0.00* | 1.00 | 1.00 | 1.00 | 1.00 |
|  | both |  | 1.00 | 0.86 | 1.00 | 0.00* | 1.00 | 1.00 | 1.00 | 1.00 |
|  | non-COVID-19 | T4: late recovery | 0.81 | 0.86 | 1.00 | 0.00* | 0.90 | 0.94 | 0.89 | 1.00 |
|  | COVID-19 |  | 0.75 | 0.86 | 0.83 | 0.00* | 0.88 | 1.00 | 0.67 | 1.00 |
|  | both |  | 0.94 | 0.76 | 0.92 | 0.00* | 0.94 | 0.88 | 1.00 | 1.00 |
|  | non-COVID-19 | T5: late recovery | 0.81 | 0.95 | 0.89 | 0.00* | 0.90 | 0.94 | 1.00 | 1.00 |
|  | COVID-19 |  | 0.88 | 0.86 | 0.33 | 0.00* | 0.88 | 0.88 | 1.00 | 0.83 |
|  | both |  | 0.94 | 0.76 | 0.87 | 0.00* | 1.00 | 0.69 | 1.00 | 0.83 |
| COVID-19 associated sepsis *vs* non-COVID-19-associated sepsis | | T1: acute | 0.78 | 0.75 | 0.50 | 0.43 | 0.76 | 0.69 | 0.63 | 0.89 |
|  |  | T2: subacute | 0.72 | 0.60 | 1.00 | 0.71 | 0.80 | 0.36 | 0.67 | 0.44 |
|  |  | T4: late recovery | 0.81 | 0.94 | 0.83 | 0.86 | 0.88 | 0.70 | 0.67 | 0.78 |
|  |  | T5: late recovery | 0.69 | 0.88 | 0.33 | 0.67 | 0.88 | 0.60 | 0.67 | 0.80 |

T1, 3±1 days after sepsis onset; T2, 7±1 days after sepsis onset; T4, 6±2 months after sepsis onset, T5, 12±2months after sepsis onset

*specificity values for adult female control group are based on data from a single volunteer and cannot be interpreted as the estimated 95% CI would be at least ±0.85.

**TABLE S5** Stability of the models over two groups with different BMI. The color fill highlights low values for visualization purposes. Specificity (Spec) values remain slightly lower for sepsis detection within BMI < 25 kg/m² group, suggesting the possibility of impact of BMI on the sepsis detection models. However, the low sample size in these subgroups might also explain the difference.

|  | | | BMI < 25 kg/m² | | BMI ≥ 25 kg/m² | |
| --- | --- | --- | --- | --- | --- | --- |
| **Task / Sepsis type** | | **Phase** | **Sens** | **Spec** | **Sens** | **Spec** |
| patients with sepsis vs healthy | non-COVID-19 | T1: acute | 1.00 | 0.95 | 0.94 | 1.00 |
|  | COVID-19 |  | 1.00 | 0.90 | 1.00 | 1.00 |
|  | both |  | 1.00 | 0.90 | 1.00 | 1.00 |
|  | non-COVID-19 | T2: subacute | 0.90 | 0.90 | 1.00 | 1.00 |
|  | COVID-19 |  | 1.00 | 0.90 | 0.98 | 1.00 |
|  | both |  | 1.00 | 0.86 | 1.00 | 0.96 |
|  | non-COVID-19 | T4: late recovery | 0.88 | 0.76 | 0.88 | 1.00 |
|  | COVID-19 |  | 1.00 | 0.86 | 0.76 | 0.96 |
|  | both |  | 0.85 | 0.76 | 0.98 | 0.87 |
|  | non-COVID-19 | T5: late recovery | 0.75 | 0.90 | 0.96 | 0.96 |
|  | COVID-19 |  | 0.67 | 0.71 | 0.80 | 0.96 |
|  | both |  | 0.89 | 0.62 | 0.96 | 0.83 |
| COVID-19 associated sepsis vs non-COVID-19-associated sepsis | | T1: acute | 0.67 | 0.70 | 0.72 | 0.71 |
|  |  | T2: subacute | 0.83 | 0.60 | 0.78 | 0.50 |
|  |  | T4: late recovery | 0.75 | 1.00 | 0.83 | 0.73 |
|  |  | T5: late recovery | 0.67 | 0.75 | 0.67 | 0.75 |

T1, 3±1 days after sepsis onset; T2, 7±1 days after sepsis onset; T4, 6±2 months after sepsis onset, T5, 12±2months after sepsis onset

References

[1] Ramoji, A., Thomas-Rüddel, D., Ryabchykov, O., Bauer, M., Arend, N., Giamarellos-Bourboulis, E.J., Eugen-Olsen, J., Kiehntopf, M., Bocklitz, T., Popp, J., Bloos, F., and Neugebauer, U., *Critical care explorations*, Vol. 3, e0394, 2021.

[2] Schie, I.W., Rüger, J., Mondol, A.S., Ramoji, A., Neugebauer, U., Krafft, C., and Popp, J., *Analytical chemistry*, Vol. 90, 2023–2030, 2018.

[3] Ryabchykov, O., Bocklitz, T., Ramoji, A., Neugebauer, U., Foerster, M., Kroegel, C., Bauer, M., Kiehntopf, M., and Popp, J., *Chemometrics and Intelligent Laboratory Systems*, Vol. 155, 1–6, 2016.
