# Supplementary material for: Raman Spectroscopy Can Identify Acute and Persistent Biochemical Changes in Leukocytes From Patients With COVID‐19 and Non‐COVID‐19‐Associated Sepsis
Source: Biotechnol J. 2025 Sep 1;20(9):e70105. doi: 10.1002/biot.70105 (PMC12402750; doi:10.1002/biot.70105)
Supplement: Supplementary file 2 — Supporting File 2: biot70105‐sup‐0001‐FigureS1.pptx. [file BIOT-20-e70105-s005.pptx]

## Slide 1
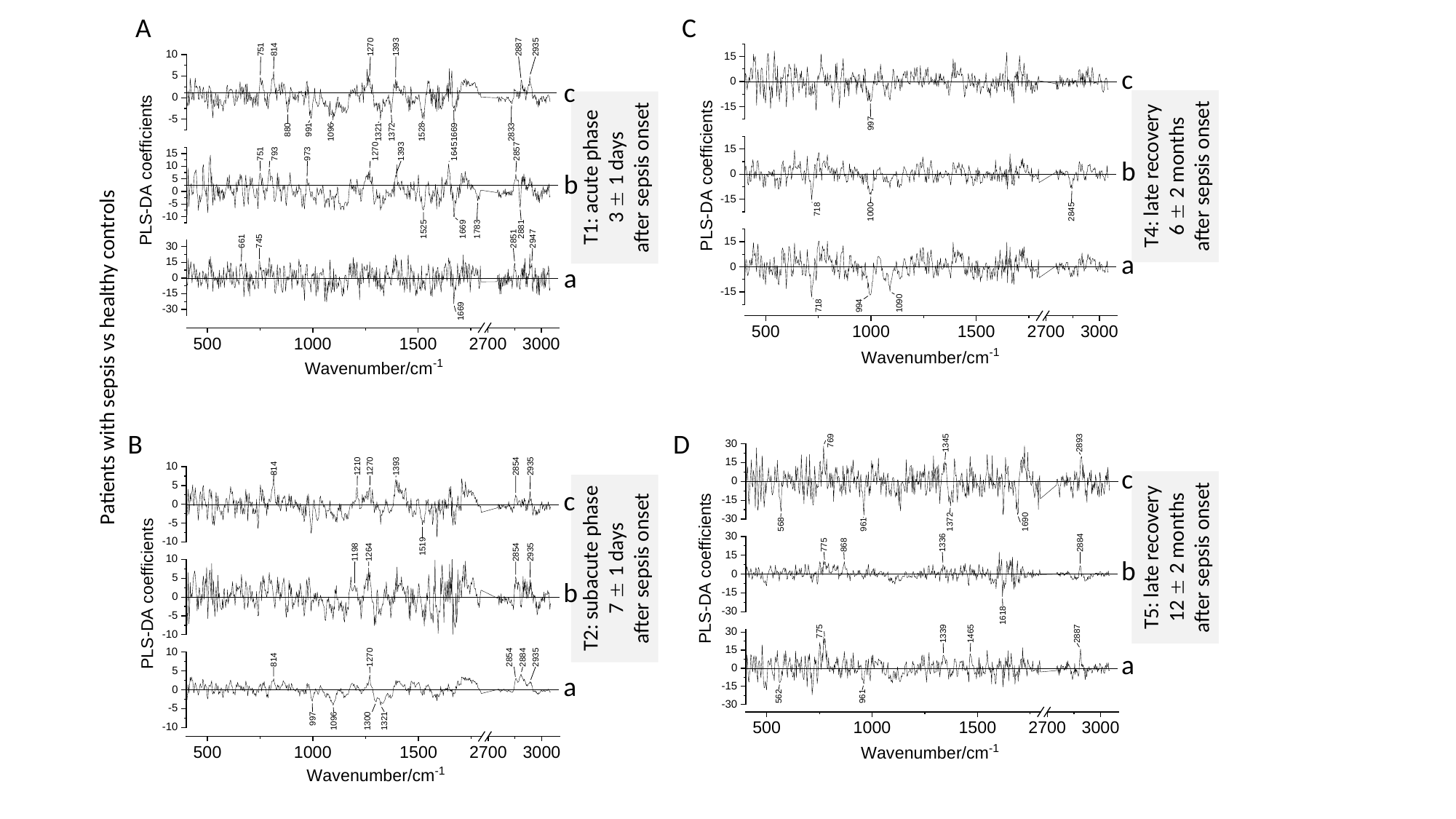

A
C
c
c
T4: late recovery
6 ± 2 months
after sepsis onset
T1: acute phase
3 ± 1 days
after sepsis onset
b
b
a
a
Patients with sepsis vs healthy controls
B
D
c
c
T5: late recovery
12 ± 2 months
after sepsis onset
T2: subacute phase
7 ± 1 days
after sepsis onset
b
b
a
a
