# Supplementary material for: Raman Spectroscopy Can Identify Acute and Persistent Biochemical Changes in Leukocytes From Patients With COVID‐19 and Non‐COVID‐19‐Associated Sepsis
Source: Biotechnol J. 2025 Sep 1;20(9):e70105. doi: 10.1002/biot.70105 (PMC12402750; doi:10.1002/biot.70105)
Supplement: Supplementary file 3 — Supporting File 3: biot70105‐sup‐0002‐FigureS2.pptx. [file BIOT-20-e70105-s006.pptx]

## Slide 1
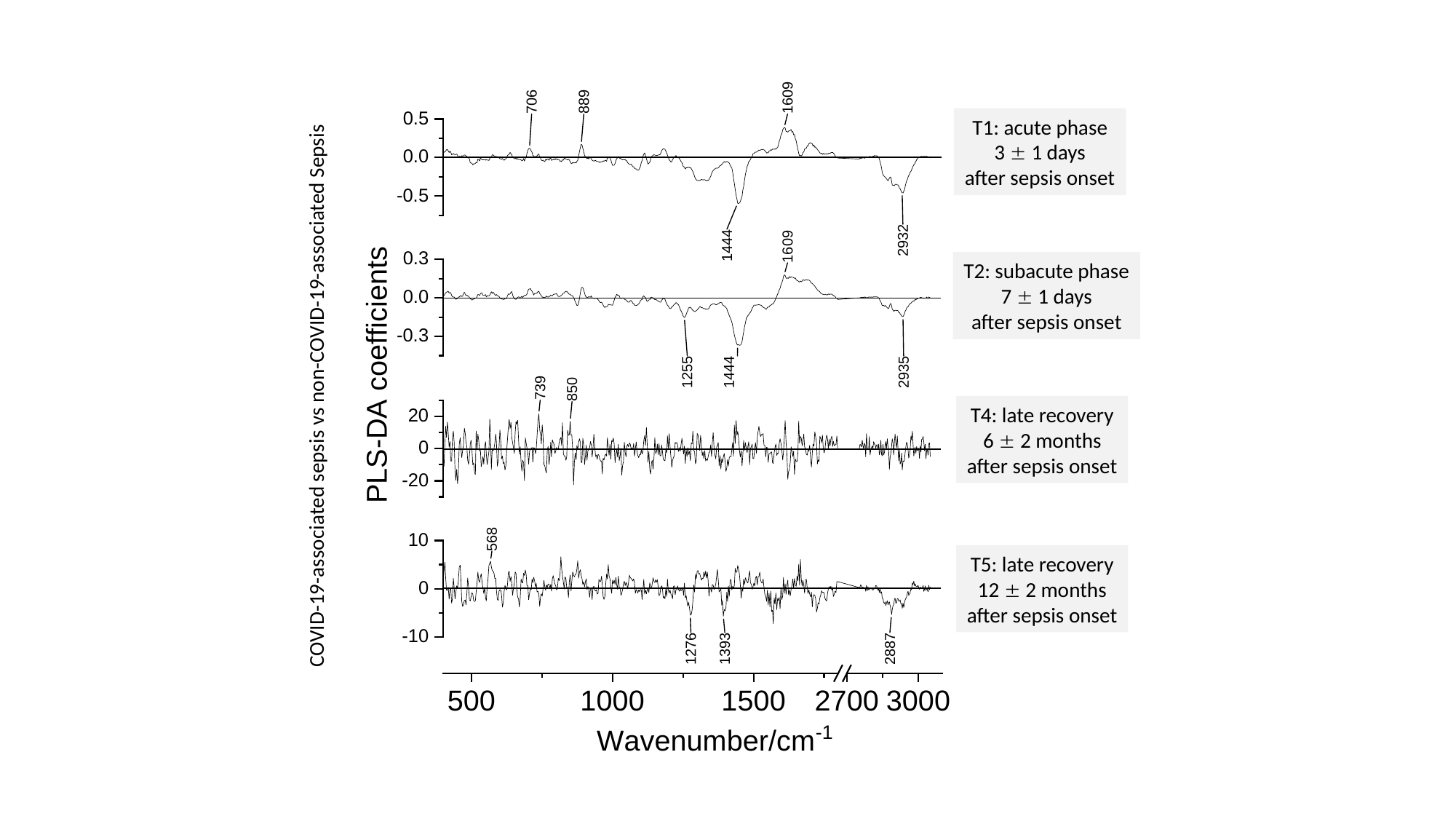

T1: acute phase
3 ± 1 days
after sepsis onset
T2: subacute phase
7 ± 1 days
after sepsis onset
COVID-19-associated sepsis vs non-COVID-19-associated Sepsis
T4: late recovery
6 ± 2 months
after sepsis onset
T5: late recovery
12 ± 2 months
after sepsis onset
