# Supplementary material for: Raman Spectroscopy Can Identify Acute and Persistent Biochemical Changes in Leukocytes From Patients With COVID‐19 and Non‐COVID‐19‐Associated Sepsis
Source: Biotechnol J. 2025 Sep 1;20(9):e70105. doi: 10.1002/biot.70105 (PMC12402750; doi:10.1002/biot.70105)
Supplement: Supplementary file 4 — Supporting File 4: biot70105‐sup‐0003‐FigureS3.pptx. [file BIOT-20-e70105-s002.pptx]

## Slide 1
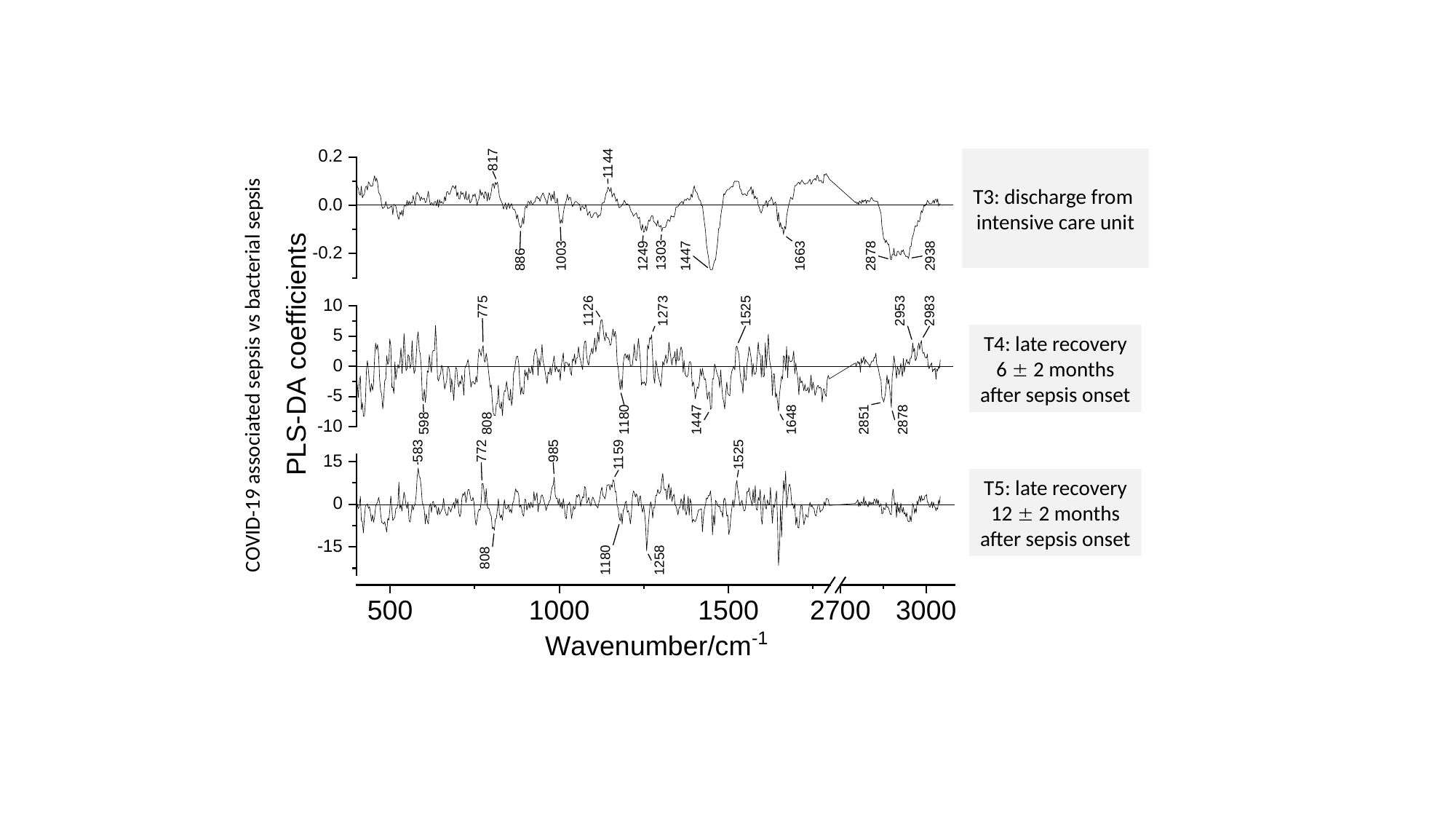

T3: discharge from
intensive care unit
T4: late recovery
6 ± 2 months
after sepsis onset
COVID-19 associated sepsis vs bacterial sepsis
T5: late recovery
12 ± 2 months
after sepsis onset
