# Supplementary figures and images for: Raman Spectroscopy Can Identify Acute and Persistent Biochemical Changes in Leukocytes From Patients With COVID‐19 and Non‐COVID‐19‐Associated Sepsis
Source: Biotechnol J. 2025 Sep 1;20(9):e70105. doi: 10.1002/biot.70105 (PMC12402750; doi:10.1002/biot.70105)

## Slide 1
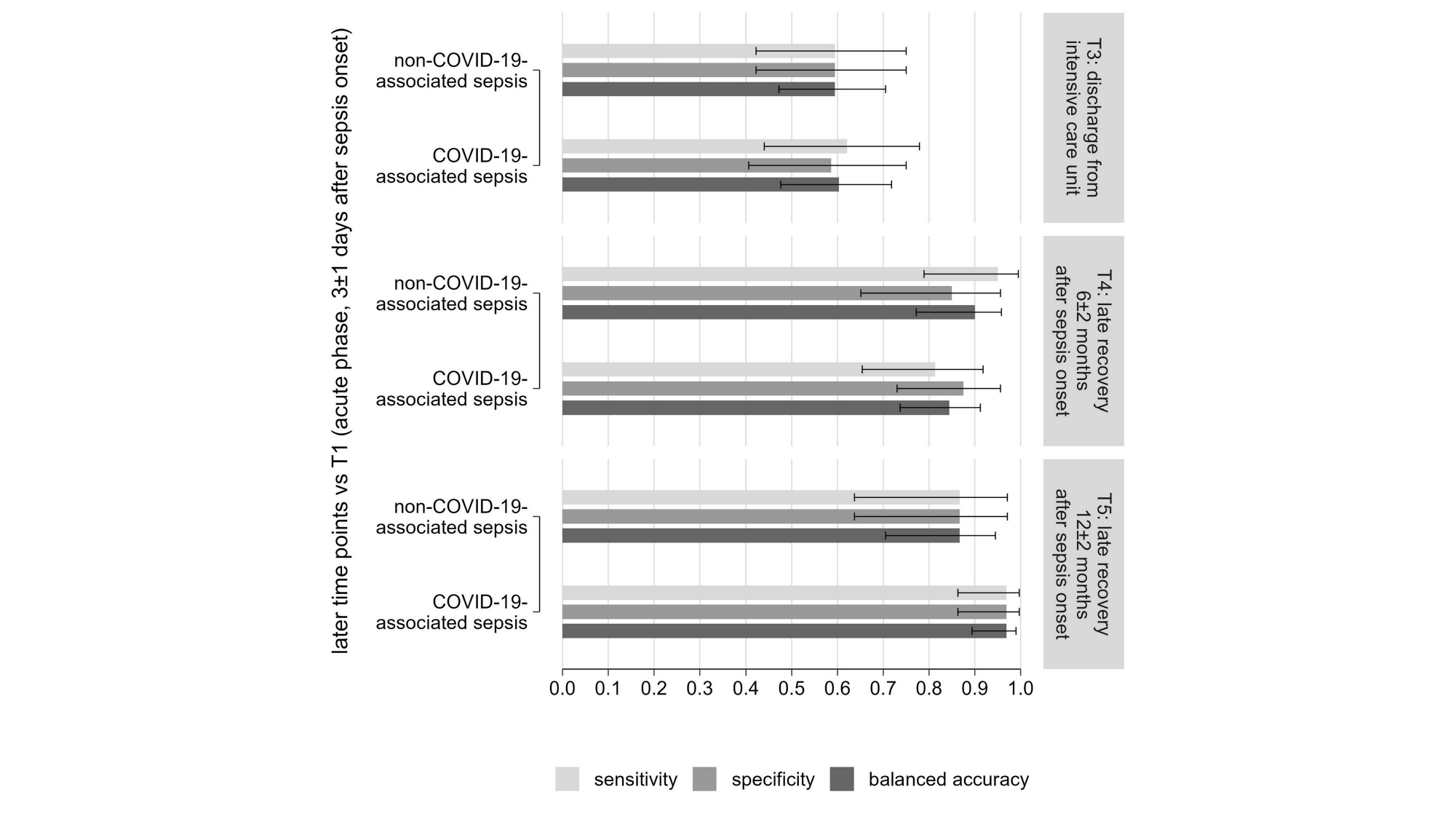

Supplement: Supplementary file 5 — Supporting File 5: biot70105‐sup‐0004‐FigureS4.pptx. [file BIOT-20-e70105-s003.pptx]
